# *Mycobacterium tuberculosis* Eis Regulates Autophagy, Inflammation, and Cell Death through Redox-dependent Signaling

**DOI:** 10.1371/journal.ppat.1001230

**Published:** 2010-12-16

**Authors:** Dong-Min Shin, Bo-Young Jeon, Hye-Mi Lee, Hyo Sun Jin, Jae-Min Yuk, Chang-Hwa Song, Sang-Hee Lee, Zee-Won Lee, Sang-Nae Cho, Jin-Man Kim, Richard L. Friedman, Eun-Kyeong Jo

**Affiliations:** 1 Department of Microbiology, College of Medicine, Chungnam National University, Daejeon, Korea; 2 Infection Signaling Network Research Center, College of Medicine, Chungnam National University, Daejeon, Korea; 3 Department of Microbiology and Brain Korea 21 Project for Medical Sciences, Yonsei University College of Medicine, Seoul, Korea; 4 Department of Pathology, College of Medicine, Chungnam National University, Daejeon, Korea; 5 Division of Life Science, Korea Basic Science Institute, Daejeon, Korea; 6 Department of Immunobiology, University of Arizona, Tucson, Arizona, United States of America; University of New Mexico, United States of America

## Abstract

The “enhanced intracellular survival” (*eis*) gene of *Mycobacterium tuberculosis* (Mtb) is involved in the intracellular survival of *M. smegmatis*. However, its exact effects on host cell function remain elusive. We herein report that Mtb Eis plays essential roles in modulating macrophage autophagy, inflammatory responses, and cell death via a reactive oxygen species (ROS)-dependent pathway. Macrophages infected with an Mtb *eis*-deletion mutant H37Rv (Mtb-*Δeis*) displayed markedly increased accumulation of massive autophagic vacuoles and formation of autophagosomes *in vitro* and *in vivo*. Infection of macrophages with Mtb-*Δeis* increased the production of tumor necrosis factor-α and interleukin-6 over the levels produced by infection with wild-type or complemented strains. Elevated ROS generation in macrophages infected with Mtb-*Δeis* (for which NADPH oxidase and mitochondria were largely responsible) rendered the cells highly sensitive to autophagy activation and cytokine production. Despite considerable activation of autophagy and proinflammatory responses, macrophages infected with Mtb-*Δeis* underwent caspase-independent cell death. This cell death was significantly inhibited by blockade of autophagy and c-Jun N-terminal kinase-ROS signaling, suggesting that excessive autophagy and oxidative stress are detrimental to cell survival. Finally, artificial over-expression of Eis or pretreatment with recombinant Eis abrogated production of both ROS and proinflammatory cytokines, which depends on the *N*-acetyltransferase domain of the Eis protein. Collectively, these data indicate that Mtb Eis suppresses host innate immune defenses by modulating autophagy, inflammation, and cell death in a redox-dependent manner.

## Author Summary

Tuberculosis is a global health problem: at least one-third of the world's population is infected with *Mycobacterium tuberculosis* (Mtb). Mtb is a successful pathogen that enhances its own intracellular survival by arresting phagolysosomal fusion. Recently, autophagy has emerged as a host defense strategy against Mtb infection, through stimulation of the fusion of phagosomes and lysosomes. However, excessive and uncontrolled autophagic activity can be detrimental to host cells and can result in their death. The Mtb “enhanced intracellular survival” (*eis*) gene has been implicated in the intracellular survival of *M. smegmatis*. However, its exact role and how it regulates host innate immune responses have not been fully explained. Here, we provide evidence that Eis suppresses macrophage autophagy, inflammation, and cell death through the inhibition of reactive oxygen species (ROS) generation. Although it has previously been demonstrated that autophagy is a key host defense response to mycobacterial infections, our data indicate that excessive autophagy, and the resulting cell death, do not significantly affect host defense responses to mycobacteria. Additionally, our data reveal that Eis's ability to regulate ROS generation and proinflammatory responses depends on its *N*-acetyltransferase domain. These results underscore a previously unrecognized role of Eis in modulating host inflammatory responses, oxidative stress, and cell survival/death during mycobacterial infection.

## Introduction

*Mycobacterium tuberculosis* (Mtb) is an intracellular pathogen that can survive and even multiply within host macrophages [1], [2]. Mtb can persist within phagosomes by interfering with intracellular membrane trafficking and by arresting phagosome maturation in infected host cells [3]. Pathogenic mycobacteria have developed several strategies for surviving and growing under nutrient-limited conditions [4]. *Autophagy*, or the removal of aged organelles, plays a central role in regulating important cellular functions [5], [6] and aids in innate and adaptive immune defense against Mtb and other intracellular pathogens [5], [7]–[9]. Physiological or pharmacological induction of autophagy in macrophages results in increased co-localization of mycobacterial phagosomes and the autophagy effector LC3, and the fusion of the former with lysosomes, which overcomes the blockade of membrane trafficking and increased bactericidal activity [7].

Although autophagy plays key roles in host innate and adaptive immune defenses, it can, under certain circumstances, result in type II programmed cell death [10], [11]. Autophagic processes are activated in response to cellular stresses, such as oxidative stress, and can influence several types of cell death, including autophagy-related cell death [12]. Recently, we showed that the mycobacterial BCG cell wall triggers autophagy-induced cell death in radiosensitized colon cancer cells [13]. Additionally, several viral gene products may be involved in autophagy-induced cell death [14]. However, the genetic basis for mycobacterial induction of autophagy, and its implications for host cell viability, remain to be elucidated.

The “enhanced intracellular survival” (*eis*) gene and its protein product, Eis, a unique protein of 42 kDa, of Mtb H37Rv enhance the survival of the saprophytic *M. smegmatis* during repeated passage through the human macrophage-like cell line U-937 [15]. Bioinformatic analyses showed that Eis is a member of the GCN5-related family of *N*-acetyltransferases [16]. Recent studies have revealed that kanamycin resistance is associated with *eis* promoter mutations that increase Eis transcript and protein levels [17]. Additionally, regulation of *eis* expression by SigA enhanced intracellular growth of the W-Beijing Mtb strain in monocytic cells [18]. Moreover, Eis inhibited the proliferation of mitogen-activated T cells and, by blocking the phosphorylation of extracellular signal-regulated kinase (ERK), reduced the production of tumor necrosis factor (TNF)-α and interleukin (IL)-4 [19]. Despite being implicated in host-pathogen interactions during Mtb infection, the precise role of Eis in innate immune regulation remains to be determined.

In an effort to gain further insight into the role of Eis in host responses, we examined autophagy, inflammatory cytokine production, and reactive oxygen species (ROS) generation in macrophages infected with wild-type (Mtb-WT), *eis*-deletion (Mtb-*Δeis*), or complemented (Mtb-*c*-*eis*) Mtb strains. Infection with Mtb-*Δeis* significantly increased autophagy, inflammatory responses, and ROS generation in macrophages. NADPH oxidase (NOX) and mitochondria were found to be the major sources of ROS, which contributed to the induction of autophagy and inflammatory responses in Mtb-*Δeis*-infected cells. Increased and excessive activation of autophagy in macrophages infected with Mtb-*Δeis* had no effect on antimicrobial responses, but stimulated caspase-independent cell death (CICD). Mtb-*Δeis*-induced host cell death was regulated by autophagic pathways and influenced by c-Jun N-terminal kinase (JNK)-dependent ROS generation. Furthermore, we show that the *N*-acetyltransferase domain of Eis is responsible for its modulation of ROS generation and proinflammatory responses in macrophages.

## Results

### *Mycobacterium tuberculosis* Eis Inhibits Autophagy in Macrophages

**Figure 1:**
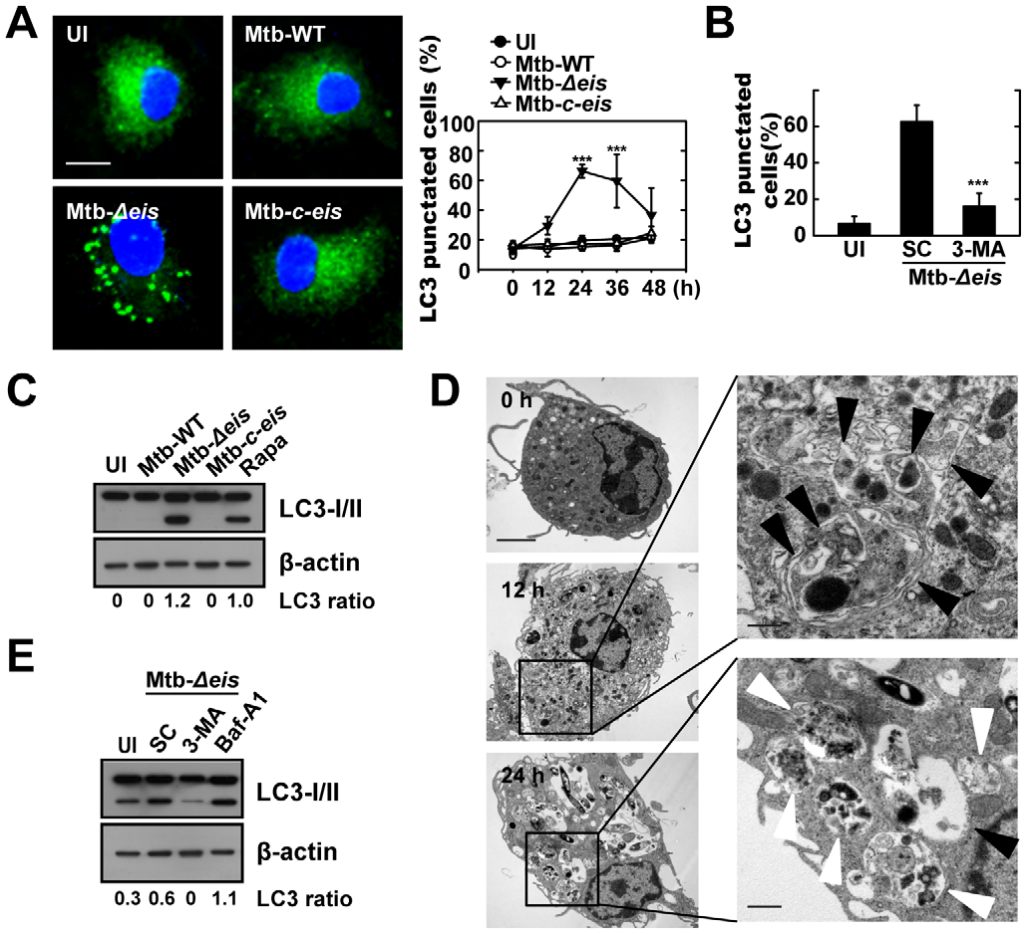
Mtb Eis modulates autophagy in macrophages. (A) BMDMs were infected with Mtb-WT, Mtb-*Δeis*, or Mtb-*c*-*eis* (MOI = 10) for 4 h (as described in the Materials and Methods), and then incubated for 24 h (*left*) or the indicated periods of time (*right*). Cells were fixed, stained with DAPI to visualize nuclei (blue), and immunolabeled with an anti-LC3 antibody. Primary antibody was detected using an Alexa Fluor 488-conjugated goat anti-rabbit IgG (green). *Left:* representative immunofluorescence images of LC3 punctae; *right:* quantification of data (LC3-punctated cells were counted manually). ***p<0.001, vs. Mtb-WT-infected condition. Scale bars, 5 µm. (B) BMDMs were infected with Mtb-*Δeis* in the absence or presence of 3-methyladenine (3-MA; 10 mM) and subjected to confocal analysis as described in Figure 1A. LC3-punctated cells were counted manually. Each condition was assayed in triplicate, and at least 250 cells were counted in each well. ***p<0.001, vs. SC. (C) Immunoblot analyses performed using Abs raised to LC3 or β-actin. Experimental conditions were identical to those outlined in panel A. Gel images representative of three experiments are shown. (D) Electron micrographs of Mtb-*Δeis*-infected BMDMs under low (*left*) and high (*right*) magnification show the accumulation of autophagic vesicles (black arrow, initial autophagic vacuoles; white arrow, degradative autophagic vacuoles). Scale bars: 2 µm (*left*), 0.5 µm (*right*). (E) Immunoblot analyses performed using Abs raised to LC3 or β-actin. BMDMs were infected with Mtb-*Δeis* in the presence or absence of 3-MA (10 mM) or bafilomycin A1 (Baf-A1; 100 nM). Gel images representative of three experiments are shown. The ratio of the intensities of the LC3-II/LC3-I and β-actin bands is indicated below each lane (C and E). UI, uninfected; SC, solvent control (0.1% distilled water (B), 0.1% DMSO (E)).

Previous studies identified a role for the *eis* gene in enhancing the survival of mycobacteria in human monocytic cells [15]. However, the role of *eis* in autophagy activation in macrophages, which plays a key role in defense and cellular homeostasis [5], is not fully understood. We first infected bone marrow-derived macrophages (BMDMs) with the Mtb-WT, Mtb-*Δeis*, and Mtb-*c-eis* strains of Mtb H37Rv and examined the kinetics of autophagosome formation by immunostaining for LC3. As shown in Figure 1A, in BMDMs infected with Mtb-*Δeis* we observed the recruitment of endogenous LC3 in punctate structures the formation of which peaked 24 h after infection, before decreasing substantially by 48 h post-infection (Fig. 1A, *right*). In contrast, autophagosome formation was not increased in BMDMs infected with Mtb-WT or Mtb-*c-eis* (Fig. 1A). Additionally, RAW 264.7 macrophages transfected with green fluorescent protein (GFP) fused to the autophagosome protein LC3 (GFP-LC3) [20] showed a significant increase in GFP-LC3 puncta formation when infected with Mtb-*Δeis* at a multiplicity of infection (MOI) of 10 (over levels in cells infected with Mtb-WT or Mtb-*c-eis* at the same bacterial load; Fig. S1A). Moreover, Mtb-*Δeis*-induced formation of LC3 punctae in BMDMs (Fig. 1B) and RAW 264.7 cells (Fig. S1B) was abrogated by treatment for 4 h with 3-methyladenine (3-MA), a classical inhibitor of autophagy [21].

Cleavage of soluble LC3 (LC3-I) to form LC3-II, which correlates with the extent of autophagosome formation [20], was further examined by Western blotting. As shown in Figure 1C, Mtb-*Δeis* significantly induced LC3-II formation, whereas Mtb-WT and Mtb-*c-eis* did not. We next monitored Mtb-*Δeis*-induced autophagy through detection of autophagic vacuoles or organelles by transmission electron microscopy (TEM). Ultrastructural analysis of BMDMs treated with Mtb-*Δeis* for 24 h revealed the presence of multiple cytosolic autophagic vacuoles resembling autophagosomes (Fig. 1D). Additionally, TEM analyses revealed the presence of bacilli within characteristic double-membrane autophagosomes and multiple membrane structures (Fig. 1D), a pattern characteristic of the induction of autophagy and autophagic death [22]–[24]. From 12 h post-infection, we observed Mtb-*Δeis* within autophagic vacuoles (Fig. 1D, middle), which fused with multivesicular structures [25]. At 24 h post-infection, multiple late or degradative autophagic vacuoles [25] were clearly visible, in which partially degraded cytoplasmic materials and bacteria were evident (Fig. 1D, bottom).

We also examined whether autophagic vacuoles formed in cells infected with Mtb-*Δeis* were able to mature to autolysosomes [25]. Confocal analysis showed that BMDMs infected with Mtb-*Δeis* exhibited co-localization of the autophagosomal marker LC3 and the lysosomes marker Lamp-1 (Fig. S1C). We also observed that levels of LC3-II and LC3 puncta formation in Mtb-*Δeis*-infected BMDMs were increased by pretreatment with the vacuolar H^+^-ATPase inhibitor bafilomycin A (Baf-A) [20], [26] (Fig. 1E, LC3-II; Fig. S1D, LC3 puncta formation). These findings indicate that Mtb-*Δeis* induced both autophagy and autophagosome-lysosome fusion in macrophages.

### Mtb-*Δeis* Infection Up-Regulates Proinflammatory Cytokine Production and ROS Generation in BMDMs

**Figure 2:**
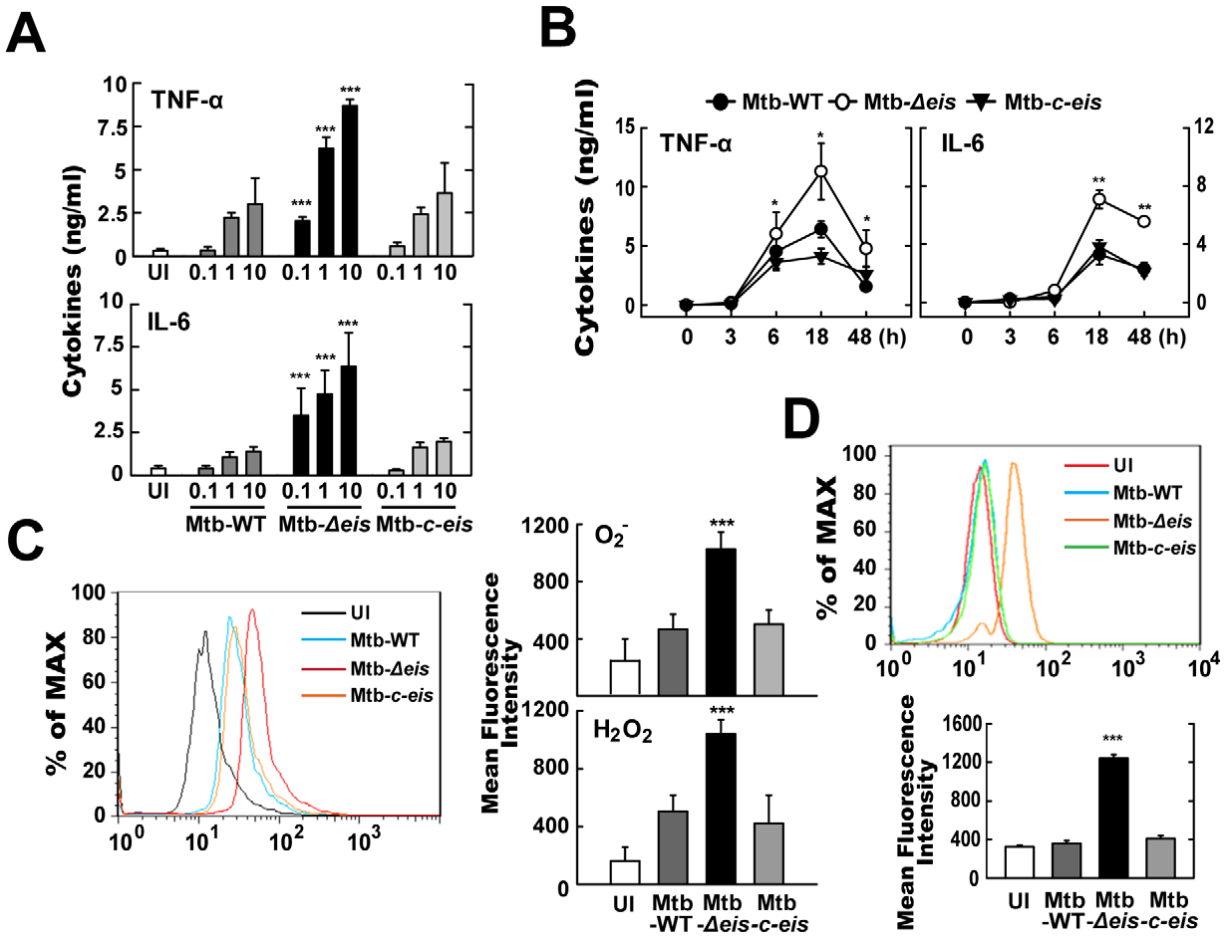
Mtb-*Δeis* infection increases production of proinflammatory cytokines and ROS by BMDMs. (A and B) BMDMs were infected with Mtb-WT, Mtb-*Δeis* or Mtb-*c*-*eis* at different MOIs (0.1, 1 and 10) for 18 h (A) or for the indicated periods of time (B; MOI = 10). Supernatants were assessed by ELISA for levels of TNF-α and IL-6. Data (A and B) are presented as the mean±SD of five experiments. (C and D) BMDMs were stimulated with Mtb-WT, Mtb-*Δeis*, or Mtb-*c*-*eis* for 30 min. Cells were then incubated with 10 µM DHE or 5 µM DCFH-DA for 15 min, washed thoroughly, and immediately analyzed for superoxide or H_2_O_2_ production by flow cytometry (C, *Left*). Cells were labeled with MitoSOX for 30 min and analyzed for mitochondrial ROS levels by flow cytometry (D, *top*). Quantitative analysis of ROS generation (C, *right*; D, *bottom*). *p<0.05, **p<0.01, ***p<0.001, vs. Mtb-WT-infected condition. UI, uninfected.

The interaction of Mtb with innate receptors in phagocytes triggers an oxidative burst and activates intracellular signaling cascades that induce proinflammatory responses [27], [28]. We thus examined the production of proinflammatory cytokines and the generation of ROS in BMDMs infected with Mtb-WT, Mtb-*Δeis*, or Mtb-*c-eis*. As shown in Figure 2A, BMDMs infected with Mtb-*Δeis* at increasing bacterial loads (MOI = 0.1, 1, 10) produced greater amounts of TNF-α and IL-6 than cells infected with Mtb-WT or Mtb-*c-eis*. Levels of TNF-α and IL-6, which peaked at 18 h, were significantly higher in BMDMs infected with Mtb-*Δeis* than those infected with Mtb-WT or Mtb-*c-eis* (Fig. 2B; *P*<0.05, TNF-α; *P*<0.01, IL-6). We next examined whether autophagy played a role in the regulation of proinflammatory cytokine production in macrophages infected with Mtb-WT, Mtb-*Δeis*, or Mtb-*c-eis*. As shown in Figure S2, the secretion of TNF-α and IL-6 was significantly increased in RAW264.7 cells transfected with siRNA specific for *Beclin-1* (siBeclin-1) or *Atg5* (siAtg5), suggesting a negative regulatory role for autophagic pathways in proinflammatory cytokine production in macrophages infected with Mtb-*Δeis*.

We further examined whether ROS levels differed between cells infected with the WT, *Δeis*, and *c-eis* strains of Mtb H37Rv. We measured the production of ROS by flow cytometry, using 2,7′-dichlorofluorescein-diacetate (DCFH-DA) and dihydroethidium (DHE) as probes for H_2_O_2_ and O_2_^−^, respectively (Fig. 2C). Compared with BMDMs infected with Mtb-WT or Mtb-*c-eis* strains, cells infected with Mtb-*Δeis* displayed markedly increased intracellular DCFH-DA and DHE fluorescence (Fig. 2C). To exclude the involvement of reactive nitrogen species (RNS) in detecting ROS generation, we pre-treated BMDMs with the specific nitric oxide synthase inhibitors nitro-L-arginine methyl ester (L-NAME) or *N* ^G^-monomethyl-L-arginine (L-NMMA) prior to Mtb-*Δeis* infection and examined ROS generation. Pre-treatment with nitric oxide synthase inhibitors had no significant effect on ROS generation in BMDMs infected with Mtb-*Δeis* (Fig. S3), suggesting that up-regulated DCFH-DA and DHE fluorescence intensities were due principally to increased ROS generation in Mtb-*Δeis*-infected macrophages. Notably, flow cytometric analysis showed that infection with Mtb-*Δeis* yielded a stronger MitoSOX Red signal, which is specific for mitochondrial superoxide [29], than infection with the Mtb-WT or Mtb-*c-eis* strains (Fig. 2D). These data suggest that Mtb-*Δeis* more strongly induces the production of proinflammatory cytokines and ROS in BMDMs than do Mtb-WT or Mtb-*c-eis*.

### ROS Generation Is Required for the Induction of Autophagy and Inflammatory Responses in Macrophages Infected with Mtb-*Δeis*

**Figure 3:**
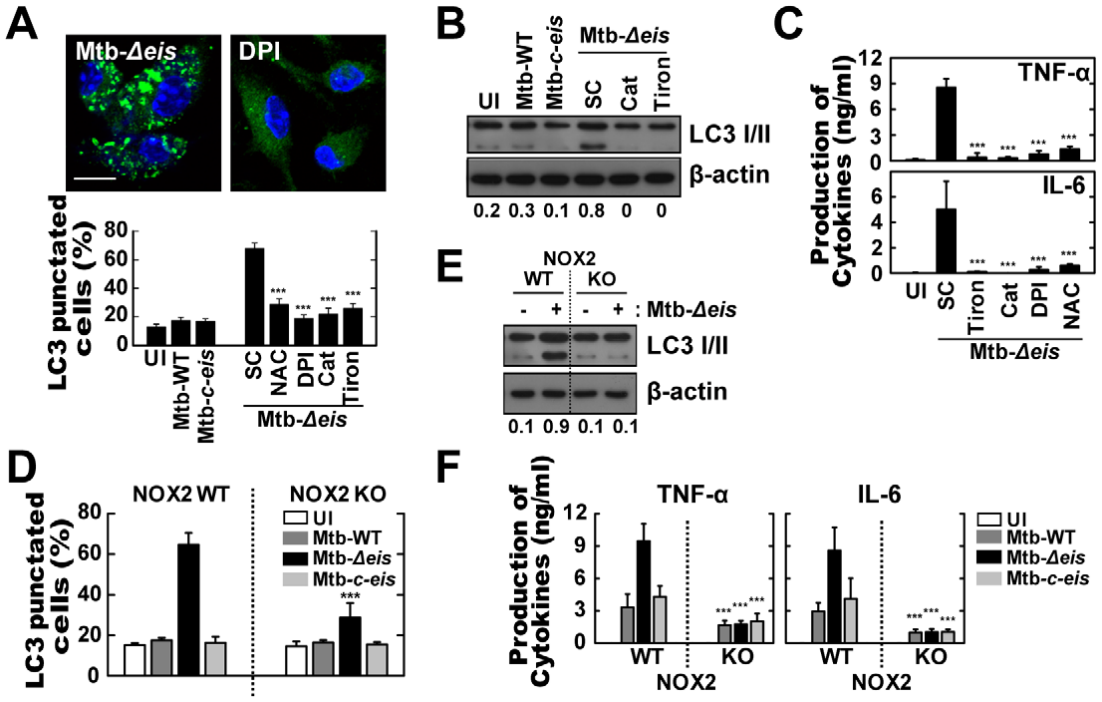
Increased ROS generation plays a critical role in autophagy and proinflammatory cytokine production in Mtb-*Δeis*-infected macrophages. (A–C) BMDMs were infected with Mtb-*Δeis* (MOI = 10) for 18 h in the presence or absence of DPI (10 µM), NAC (20 mM), catalase (Cat, 1 mU/mL), or tiron (5 mM). (A) Representative immunofluorescence images (*top*); percentage of endogenous LC3-punctated cells (*bottom*). (B) Immunoblot analyses of BMDMs with antibodies raised to LC3 or β-actin. Gel images are representative of three experiments. The ratio of the intensities of the LC3-II/LC3-I and β-actin bands is indicated below each lane. (C) Experimental conditions were identical to those outlined in Figure 3A. Supernatants collected 18 h after infection were assessed for cytokine levels by ELISA. Data represent the mean±SD of five experiments. (D–F) BMDMs from WT and NOX2 KO mice were infected with Mtb-WT, Mtb-*Δeis*, or Mtb-*c*-*eis* for 18 h. (D) Numbers of LC3-punctated cells (counted manually) are shown (at least 250 cells were counted in each well). (E) Immunoblot analyses performed using Abs raised to LC3 or β-actin. BMDMs from WT and NOX2 KO mice were infected with Mtb-*Δeis* for 18 h. Gel images representative of three experiments are shown. (F) Supernatants collected 18 h after infection were assessed for cytokine levels by ELISA. Data are presented as the mean±SD of at least three separate experiments, each performed in triplicate. ***p<0.001, vs. SC (A and C); WT mice (D and F). UI, uninfected; SC, solvent control (0.1% DMSO).

Recent studies have shown that NOX-derived ROS are involved in the activation of autophagy [30]. Additionally, we have shown that NOX2/gp91phox, the main catalytic component of NOX, interacts with TLR2, a key effector of Mtb-induced proinflammatory responses [28]. Because ROS generation was significantly elevated in Mtb-*Δeis*-infected cells, we hypothesized that increased ROS production during Mtb-*Δeis* infection might be a trigger for autophagy activation and proinflammatory responses. As anticipated, pretreatment with the ROS scavengers [*N*-acetyl cysteine (NAC), diphenyleneiodonium (DPI), catalase and tiron (4,5-dihydroxy-1,3-benzene disulfonic acid-disodium salt); for 1 h before infection] prevented Mtb-*Δeis*-induced autophagosome accumulation in BMDMs (Fig. 3A) and RAW 264.7 cells transfected with GFP-LC3 (Fig. S4A). Additionally, the conversion of LC3-I to LC3-II in Mtb-*Δeis*-infected cells was suppressed by catalase and tiron (Fig. 3B). We further examined whether ROS generation was involved in the induction of proinflammatory cytokines in Mtb-*Δeis*-infected BMDMs. ROS scavengers reduced the TNF-α and IL-6 levels in BMDMs infected with Mtb-*Δeis* (Fig. 3C).

We next determined the Mtb-*Δeis*-induced activation of autophagy and proinflammatory responses in NOX2-deficient macrophages. ROS induction was abolished in NOX2-deficient macrophages infected with Mtb-WT, Mtb-*Δeis*, or Mtb-*c-eis* (Fig. S4B). Infection of NOX2-deficient BMDMs with Mtb-*Δeis* resulted in a dramatic reduction in autophagy, as assessed by LC3 puncta formation (Fig. 3D) and LC3-II conversion (Fig. 3E) at 18 h. However, neither starvation- nor rapamycin-induced autophagy was dependent on NOX2 expression (Fig. S4C). Proinflammatory cytokine mRNA expression at 6 h (Fig. S4D) and protein levels at 18 h (Fig. 3F) following infection with Mtb-*Δeis* were significantly reduced in BMDMs taken from NOX2 KO mice. The release of proinflammatory cytokines in response to WT or *c-eis* Mtb was similarly reduced in NOX2-deficient macrophages (Fig. 3F). Collectively, our data suggest that NOX2-derived ROS are centrally involved in the up-regulated autophagy and proinflammatory responses in BMDMs infected with Mtb-*Δeis*.

### Infection with Mtb-*Δeis* Increases Caspase-independent Cell Death

**Figure 4:**
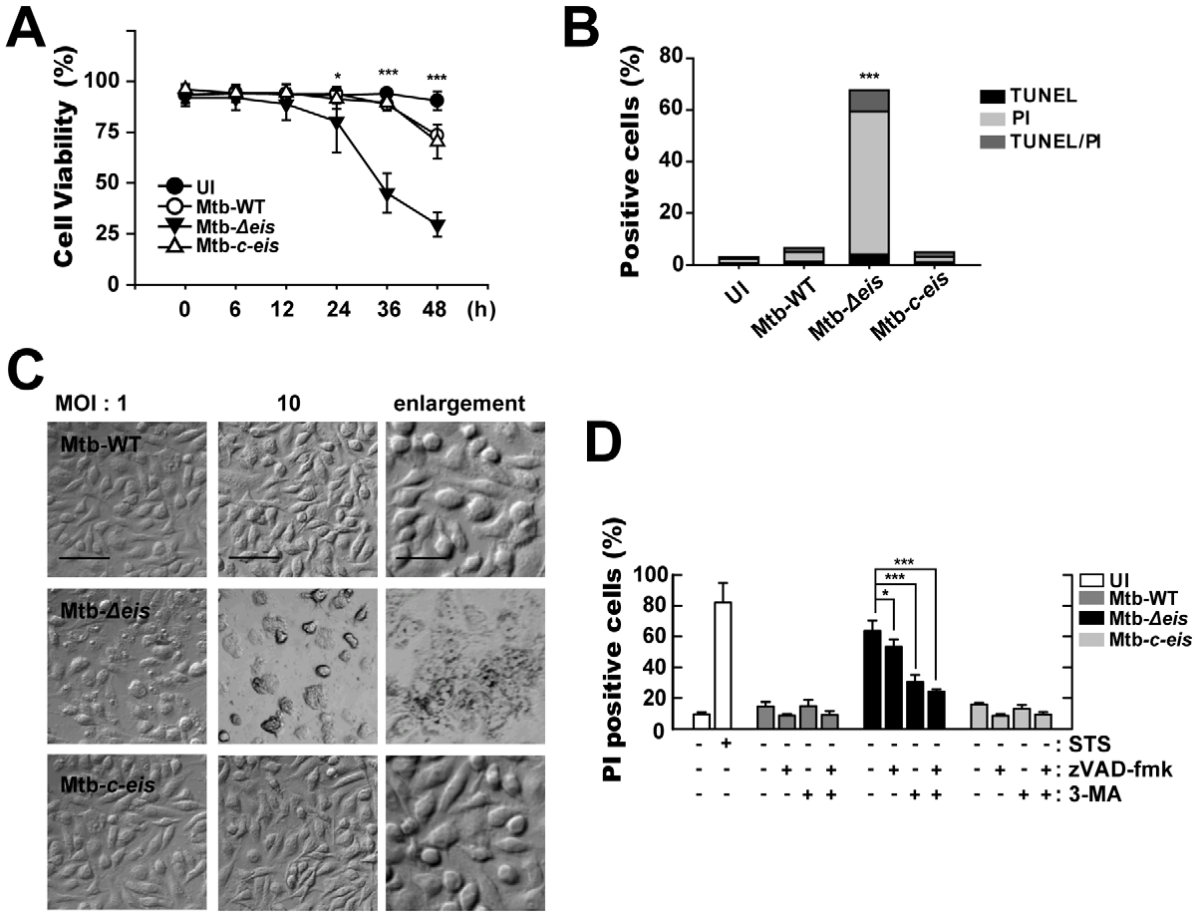
Macrophages infected with Mtb-*Δeis* show reduced cell viability and increased caspase-independent cell death. (A) BMDMs were infected with Mtb-WT, Mtb-*Δeis*, or Mtb-*c*-*eis* (MOI = 10) for the indicated periods of time, washed to remove unbound mycobacteria, and then incubated in complete DMEM at 37°C in 5% CO_2_. Cell viability was assessed by PI staining and then examined by fluorescence microscopy. (B and C) Experimental conditions were identical to those outlined in panel A. BMDMs were infected with the three strains of mycobacteria for 36 h. (B) Apoptosis was assessed using a TUNEL/apoptosis detection kit, according to the manufacturer's protocol. Cells were then examined under a laser-scanning confocal microscope (LSM 510; Zeiss). Percentages of TUNEL-positive, PI-positive, and TUNEL-/PI-double-positive cells were calculated. Data are representative of three separate experiments. (C) Morphological changes in BMDMs infected with Mtb-WT, Mtb-*Δeis*, or Mtb-*c*-*eis* at MOIs of 1 and 10. Representative images are shown. Scale bars: 50 µm (low magnification), 20 µm (high magnification). (D) Macrophages were infected with Mtb-WT, Mtb-*Δeis*, or Mtb-*c*-*eis* in the presence or absence of zVAD-fmk (20 µM) or 3-MA (10 mM). Staurosporine (STS; 500 nM) was used as a positive control. After 36 h, cells were stained with PI and then examined by fluorescence microscopy. Data are presented as the mean±SD of at least three separate experiments, each performed in duplicate. *p<0.05, ***p<0.001, vs. Mtb-WT-infected condition (A and B); Mtb-*Δeis*–infected condition without inhibitors (D). UI, uninfected.

Autophagy serves as a cell survival mechanism in some contexts, but triggers cell death in others [31]. To examine whether the *eis* gene can modulate host cell survival/death in macrophages, we infected BMDMs with Mtb-WT, Mtb-*Δeis*, or Mtb-*c-eis* and examined host cell viability. When BMDMs were infected with these three strains at an MOI of 10, Mtb-*Δeis*-infected cells showed a significant decrease in cell viability after 24 h, whereas Mtb-WT- and Mtb-*c-eis*-infected cells displayed only low rates of cell death (Fig. 4A). Infection with either Mtb-WT or Mtb-*c-eis* tended to reduce BMDM viability, dose-dependently, above an MOI of 25 (Fig. S5A).

We next assessed whether apoptosis played a role in the cell death induced by Mtb-*Δeis* using the TUNEL assay (Fig. 4B). At 36 h post-infection, there was a marked increase in total cell death in BMDMs infected with Mtb-*Δeis*. However, only a slight increase in the number of apoptotic cells was observed (Fig. 4B). Additionally, microscopic examination of Mtb-*Δeis*-infected cells at 36 h post-infection revealed morphological changes associated with cell death that were not observed in Mtb-WT- or Mtb-*c-eis*-infected cells (Fig. 4C). http://www.jimmunol.org/cgi/content/full/179/2/939 - F1#F1To further examine the mechanism of cell death in Mtb-*Δeis*-infected cells, we cultured BMDMs infected with Mtb-WT, Mtb-*Δeis*, or Mtb-*c-eis* (MOI = 10) in the presence or absence of the broad-spectrum caspase inhibitor z-VAD-fmk (administered 1 h prior to infection). We found that z-VAD-fmk only partially blocked Mtb-*Δeis*-mediated cell death (Fig. 4D). Also, caspase-3 enzyme activities did not differ significantly between Mtb-WT-, Mtb-*Δeis*-, and Mtb-*c-eis*-infected macrophages (data not shown), suggesting that the reduction in macrophage viability caused by Mtb-*Δeis* infection did not result primarily from caspase activation.

We further assessed the role of autophagy in modulating cell death induced by Mtb-*Δeis*. BMDMs were pretreated with a known inhibitor of autophagy, 3-MA, prior to Mtb-*Δeis* infection. Pretreatment with 3-MA effectively prevented Mtb-*Δeis*-induced macrophage cell death, but had no such effect on Mtb-WT- or Mtb-*c-eis*-infected cells (Fig. 4D)http://www.jimmunol.org/cgi/content/full/180/1/207 - F6#F6. To further assess the role of autophagy in Mtb-*Δeis*-induced cell death, we depleted *Beclin-1* or *Atg5* by siRNA transfection of Mtb-*Δeis*-infected RAW 264.7 cells. Transfection of RAW 264.7 cells with siBeclin-1 or siAtg5 significantly inhibited Mtb-*Δeis*-induced cell death, as assessed by propidium iodide (PI) staining (Fig. S5B). Moreover, a trypan blue exclusion assay showed that blockade of autophagy increased the survival of Mtb-*Δeis*-infected BMDMs (Fig. S5C). Collectively, these results support the concept that Mtb Eis actively inhibited CICD.

### Macrophage Death and ROS Generation Induced by Mtb-*Δeis* Depend on JNK Signaling

**Figure 5:**
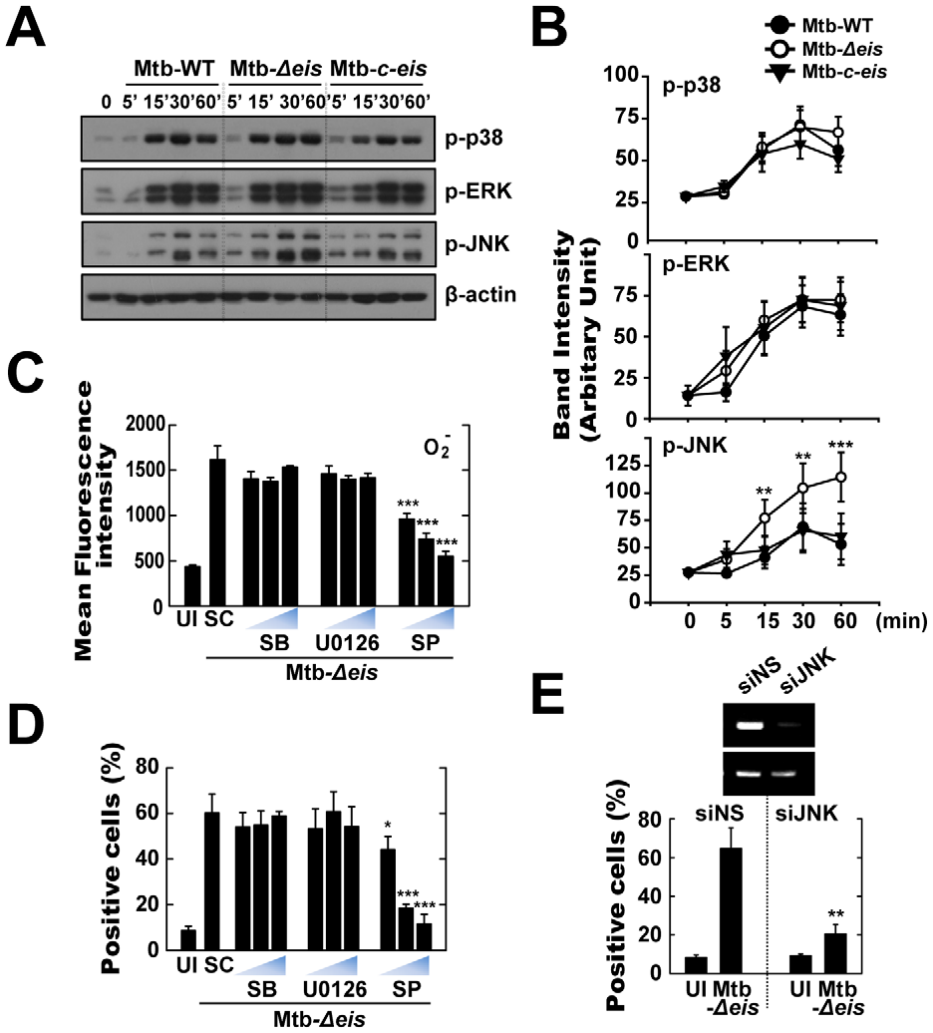
Infection with Mtb-*Δeis* induces cell death through JNK-dependent signaling. (A and B) BMDMs were infected with Mtb-WT, Mtb-*Δeis*, or Mtb-*c*-*eis* (MOI = 1) for the indicated periods of time, and then subjected to Western blot analysis using Abs raised to p-ERK1/2, p-p38, p-JNK, and β-actin. Data shown are representative of three independent experiments that all yielded similar results (A). Expression of phospho-MAPK/β-actin protein in cytoplasmic extracts of BMDMs was quantified densitometrically (B). (C and D) BMDMs were pretreated with U0126 (5, 10, 20 µM), SB203580 (SB; 1, 5, 10 µM), or SP600125 (SP; 5, 10, 20 µM) for 45 min, and then infected with Mtb-*Δeis* for 30 min (C) or 36 h (D). (C) Cells were then incubated with DHE for 15 min, washed rapidly and thoroughly, and analyzed immediately for superoxide levels by flow cytometry. Quantitative DHE fluorescence data represent the mean±SD of four experiments. (D) Cell death after 36 h was assessed by PI staining and then examined by fluorescence microscopy. (E) Raw264.7 cells were transfected with siRNA specific for JNK1 (siJNK) or a non-specific control siRNA (siNS). At 24 h after transfection, cells were infected with Mtb-*Δeis* for 36 h. Cell death was then assessed by PI staining, and then examined by flow cytometry. Transfection efficiency was assessed by RT-PCR (*inset*). Data represent the mean±SD of five random fields and are representative of three independent experiments (D and E). *p<0.05, **p<0.01, ***p<0.001, vs. Mtb-WT-infected condition (B); SC (C and D); siNS (E). UI, uninfected; SC, solvent control (0.1% DMSO).

It is known that ERK and JNK mitogen-activated protein kinase (MAPK) signaling pathways are important in oxidative stress-induced cell death [32]–[34]. No significant difference in activation kinetics of phosphorylated p38 and ERK1/2 was detected between cells infected with Mtb-WT-, Mtb-*Δeis*-, and Mtb-*c-eis* (Fig. 5A). In contrast, a significant increase in JNK/SAPK phosphorylation was observed in cells infected with Mtb-*Δeis*, this response preceding similar responses in macrophages infected with Mtb-WT or Mtb-*c-eis* (Fig. 5A). Densitometric quantification of phosphorylated p38, ERK1/2, and JNK band intensities showed that the active form of JNK was uniquely increased in macrophages infected with Mtb-*Δeis* (Fig. 5B). These results indicate that differences in JNK signaling may be responsible for differences in the responses to different bacterial strains.

To further explore the link between MAPK signaling and Mtb-*Δeis*-induced ROS generation and cell death, cells were pretreated with specific inhibitors of JNK (SP600125), p38 (SB203580), and MEK (U0126) for 1 h prior to infection with Mtb-*Δeis*. Inhibition of JNK, but not the other two kinases, dose-dependently reduced Mtb-*Δeis*-induced ROS generation, as measured by flow cytometry (Fig. 5C). Additionally, inhibition of JNK signaling, but not p38 or ERK1/2 signaling, dose-dependently reduced Mtb-*Δeis*-induced macrophage death (Fig. 5D). Moreover, transfection of RAW264.7 cells with siRNA specific for JNK (siJNK) markedly reduced cell death induced by Mtb-*Δeis* (Fig. 5E). Together, these data suggest that Eis modulated macrophage survival through JNK-dependent regulation of ROS signaling.

### Mtb-*Δeis* Increases the Accumulation of Autophagic Vesicles and Causes Lung Inflammation in Infected Mice

**Figure 6:**
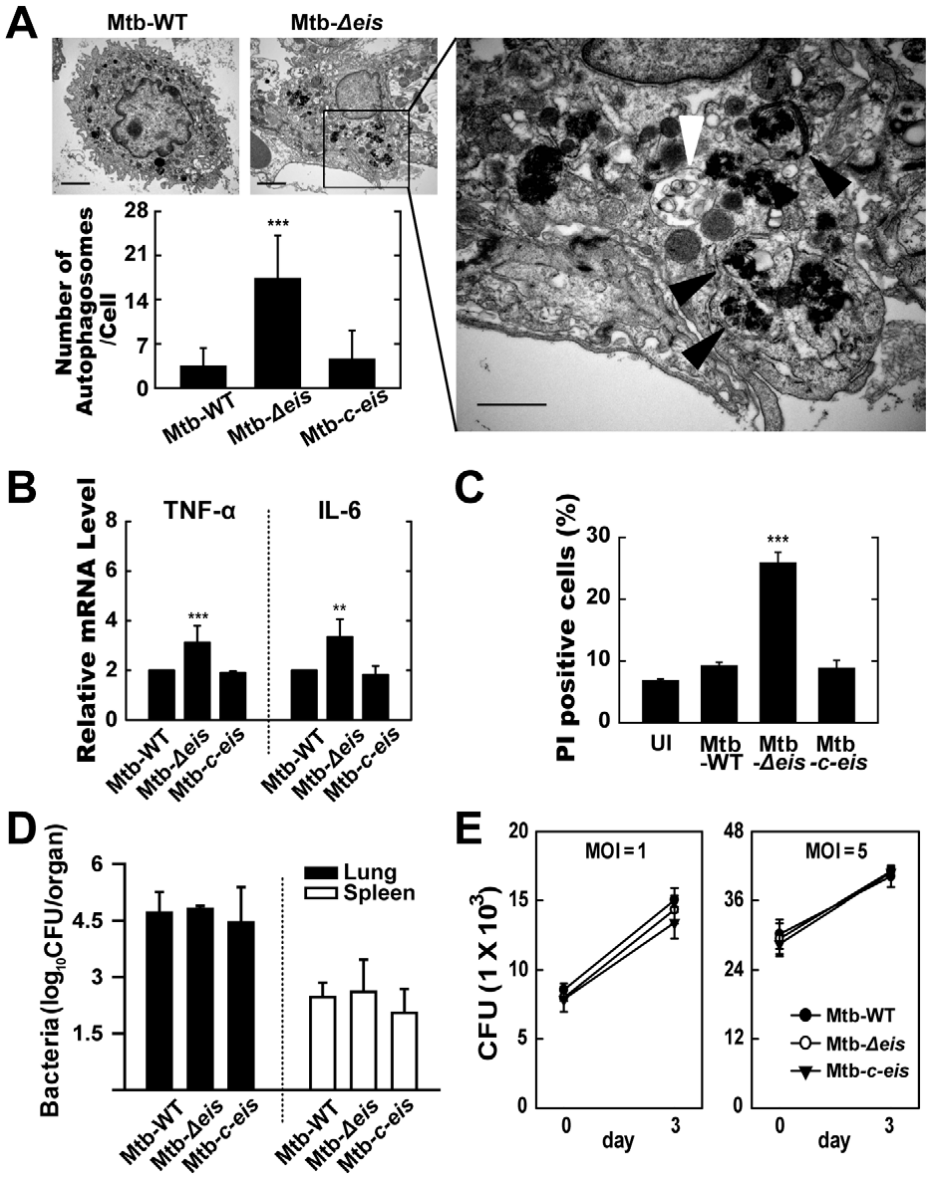
*In vivo* analysis of autophagic vesicles, inflammation, and cell death in infected mice with Mtb-*Δeis*. C57BL/6 mice were challenged, by aerosol, with 10–30 CFU Mtb-WT, Mtb-*Δeis*, or Mtb-*c*-*eis* and sacrificed 4 weeks post-infection. (A) High- and low-magnification electron micrographs of lung tissue sections from mice infected with Mtb-*Δeis* show accumulation of autophagic vesicles (black arrow, bacteria in autophagic vacuoles; white arrow, degradative autophagic vacuoles). Scale bars: 2 µm (*left upper*), 0.5 µm (*right*). Numbers of autophagic vacuoles per cell in each TEM section (*left lower*) (mean±SD; *n* = 50). (B) Quantitative RT-PCR analysis of lung tissue from Mtb-WT-, Mtb-*Δeis*-, and Mtb-*c-eis*-infected mice. Total RNA was extracted from paraffin-embedded lung tissue sections, as described in the Materials and Methods. (C) To assess *in vivo* cell death, bronchoalveolar lavage fluid cells from Mtb-WT-, Mtb-*Δeis*-, and Mtb-*c-eis*-infected mice were subjected to PI staining, and analyzed by flow cytometry. Data are presented as the mean±SEM (*n* = 4). (D) Numbers of CFUs in lung and spleen 4 weeks after infection with Mtb-WT, Mtb-*Δeis,* or Mtb-*c-eis*. Data are presented as log_10_ CFU±SEM (*n* = 4). (E) BMDMs were infected with Mtb-WT, Mtb-*Δeis*, or Mtb-*c-eis* and then analyzed by CFU assay. CFU data represent the mean±SD of four individual experiments. **p<0.01, ***p<0.001, vs. Mtb-WT-infected condition. UI, uninfected.

We next investigated the activation of autophagy, inflammation, cell death, and mycobacterial growth *in vivo*. Mice were challenged, by aerosol exposure to Mtb-WT, Mtb-*Δeis*, or Mtb-*c-eis*, and maintained for 4 weeks. Rates/levels of pulmonary granulomatous inflammation were approximately 35–50% at 4 weeks post-infection (data not shown). Similar to the *in vitro* results (Fig. 1), numerous lamellar structures with cytoplasmic autophagic vacuoles were observed in the cytosol of alveolar macrophages isolated from the lungs of mice 4 weeks after infection with Mtb-*Δeis*, but not Mtb-WT or Mtb-*c-eis* (Fig. 6A and other data not shown). These ultrastructural features demonstrated the presence and degradation of bacteria within autophagic vesicles in the lungs of Mtb-*Δeis*-infected mice (Fig. 6A). Additionally, quantitative RT-PCR analysis demonstrated that TNF-α and IL-6 mRNA levels were significantly higher in lung tissues from Mtb-*Δeis*-infected mice than in those from Mtb-WT- or Mtb-*c-eis*-infected mice (Fig. 6B). Moreover, rates of cell death, measured by PI staining, were significantly higher in bronchoalveolar lavage fluid cells isolated from Mtb-*Δeis*-infected mice than those from Mtb-WT- or Mtb-*c-eis*-infected mice (Fig. 6C). There was no significant difference in the number of TUNEL-positive apoptotic cells in lung tissues from Mtb-WT-, Mtb-*Δeis*-, and Mtb-*c-eis*-infected mice (data not shown).

To analyze bacterial survival *in vivo*, five mice per group were sacrificed 4 weeks post-challenge and bacterial counts were determined from lung and spleen homogenates. Numbers of viable bacteria in lung and spleen did not differ among mice infected with the three Mtb strains (Fig. 6D). Furthermore, we determined the *in vitro* intracellular growth of Mtb-WT, Mtb-*Δeis*, and Mtb-*c-eis* in macrophages. The three strains grew in macrophages at almost identical rates (Fig. 6E), consistent with our previous observations [16]. Collectively, these data suggest that numbers of autophagic vacuoles, the strength of the inflammatory response, and rates of cell death were significantly increased during *in vivo* infection with Mtb-*Δeis*, although there was no obvious effect on bacterial elimination.

### The *N*-acetyltransferase Domain of Eis Mediates ROS Generation and Proinflammatory Cytokine Production

**Figure 7:**
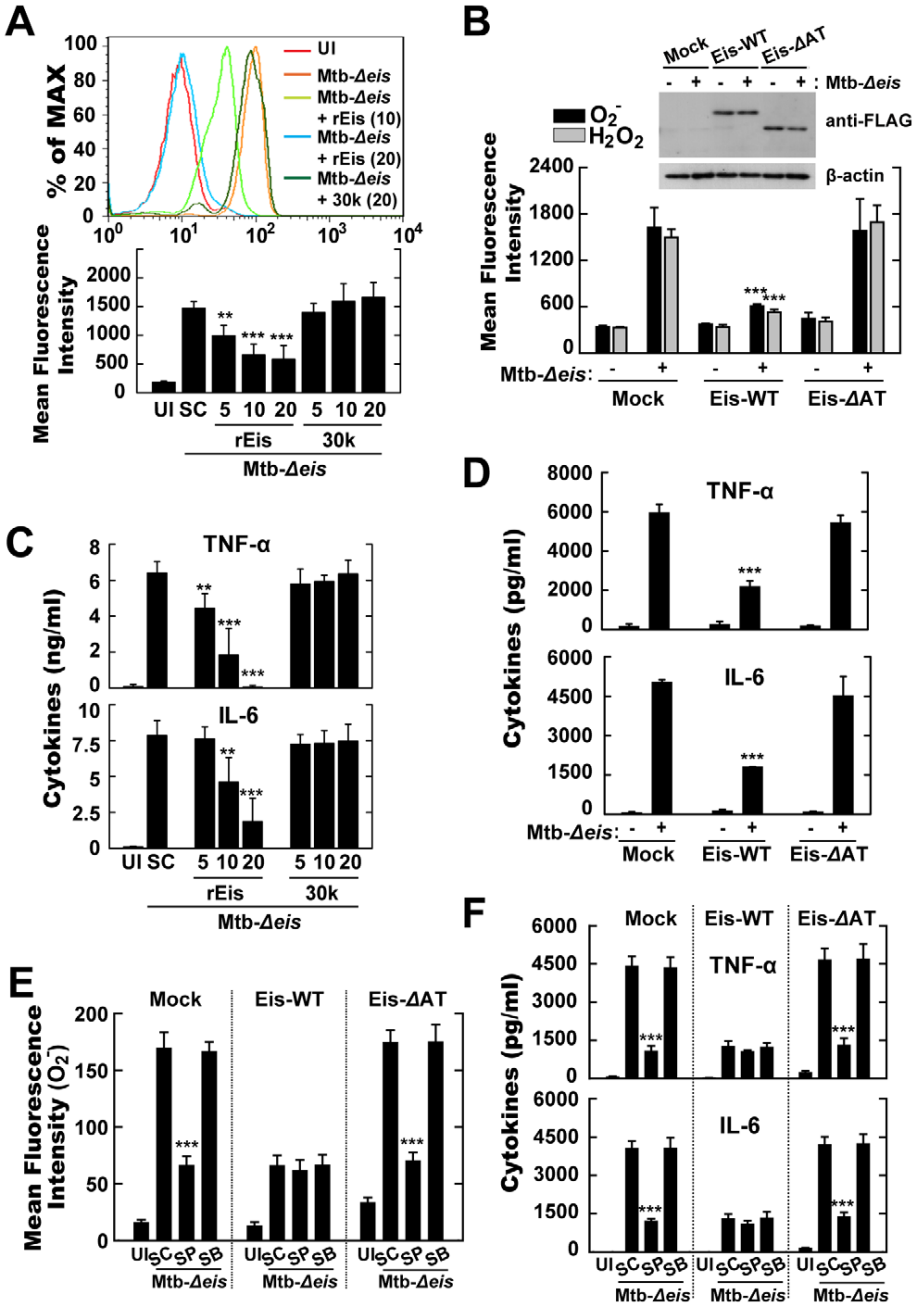
Eis modulates ROS release and proinflammatory cytokine production through its *N*-acetyltransferase domain. (A) Intracellular superoxide production was analyzed by flow cytometric analysis. BMDMs were infected with Mtb-*Δeis* (MOI = 10) in the presence or absence of recombinant Eis protein (rEis; 5, 10, 20 µg/mL) or 30 kDa Mtb antigen (30 k; 5, 10, 20 µg/mL). *Upper*, representative flow cytometric analysis; *lower*, quantitation of superoxide generation. (B) THP-1 cells were transfected with mock, Eis-WT, or Eis-*Δ*AT constructs, and infected with Mtb-*Δeis* for 30 min. Cells were stained with DHE (for superoxide) or DCFH-DA (for H_2_O_2_) and subjected to flow cytometric analysis. *Inset,* transfection efficiency. (C and D) Experimental conditions were identical to those outlined in panels A and B, respectively. Supernatants were collected 18 h after infection and assessed by ELISA for levels of TNF-α and IL-6. Data are presented as the mean±SD of five experiments. (E and F) THP-1 cells transfected with mock, Eis-WT, or Eis-*Δ*AT constructs were pretreated with SP600125 (SP; 20 µM) or SB203580 (SB; 5 µM) for 45 min before infection with Mtb-*Δeis* for 30 min (E) or 18 h (F). E, Intracellular superoxide production was analyzed by flow cytometric analysis. F, ELISA analysis for TNF-α and IL-6 levels. Data are presented as the mean±SD of three experiments. **p<0.01, ***p<0.001, vs. SC (A, C, E, and F); mock control (B and D). UI, uninfected; SC, solvent control (0.1% DMSO).

We previously showed that Mtb-infected macrophages release Eis into the cytosol and the culture supernatant [16]. Thus, the potential of recombinant Eis protein to inhibit ROS generation and inflammatory cytokine production in macrophages infected with Mtb-*Δeis* was assessed by Eis pretreatment or transfection with the *eis* gene. Induction of ROS by Mtb-*Δeis* was significantly decreased by pretreatment with Eis, but not by control mycobacterial antigens, such as the recombinant 85A (30 k) antigen of Mtb (Fig. 7A). Eis is a member of the GCN5-related family of *N*-acetyltransferases [16]. To test whether the acetyltransferase domain of Eis was required for the induction of ROS, we transfected THP-1 cells with an Eis-WT (WT-*eis*-expressing) or Eis-*Δ*AT (*N-*acetyltransferase domain deletion mutant) construct, or a mock control plasmid, and infected them with Mtb-*Δeis*. Eis-WT, but not Eis-*Δ*AT, blocked the induction of superoxide and H_2_O_2_ generation by Mtb-*Δeis* (Fig. 7B).

We next examined the effect of Eis pretreatment on the proinflammatory cytokine production in Mtb-*Δeis*-infected BMDMs. Pretreatment with Eis, but not 85A antigen, dose-dependently inhibited Mtb-*Δeis*-induced secretion of TNF-α and IL-6 (Fig. 7C). Moreover, we examined the effects of over-expressing Eis-WT or Eis-*Δ*AT plasmids on proinflammatory cytokine responses in THP-1 cells infected with Mtb-*Δeis*. Cells over-expressing wild-type Eis secreted 2.6-fold less TNF-α and 2.7-fold less IL-6 than those expressing an Eis protein lacking the AT domain (Fig. 7D). Notably, inhibition of the JNK pathway by pre-treatment with pharmacological inhibitors markedly blocked Mtb-*Δeis*-mediated up-regulation of superoxide generation (Fig. 7E) and proinflammatory cytokine levels (Fig. 7F) in THP-1 cells transfected with either mock control or Eis-*Δ*AT constructs. In contrast, JNK inhibition did not significantly affect Mtb-*Δeis*-induced ROS production (Fig. 7E) or cytokine secretion (Fig. 7F) in THP-1 cells over-expressing Eis-WT constructs. These data suggest that the *N*-acetyltransferase domain of Eis is critical to Eis's modulation of host cell ROS generation and proinflammatory cytokine responses through the JNK pathway.

## Discussion

Earlier studies demonstrated that the *eis* gene of Mtb can enhance survival of the non-pathogenic *M. smegmatis* in macrophages [15]. Moreover, Eis protein was detected in Mtb-containing phagosomes and the cytoplasm of parasitized cells, as well as in cell culture supernatants of Mtb-infected macrophages [16], [35]. Studies have demonstrated the presence of anti-Eis antibodies in TB patients, indicating that Eis is produced during human infection [35]. Eis also modulates TNF-α secretion and T cell responses [16], [19]. However, its precise role in innate immune responses has not been clearly determined. The present study provides evidence that Eis plays an essential role in modulating host innate responses and cell death through ROS-dependent pathways. Our demonstration that Mtb-*Δeis* increased the production of proinflammatory cytokines by BMDMs (i.e., Eis production alters patterns of cytokine production) is consistent with our previous findings [16]. Additionally, we provide evidence that Eis performs previously unrecognized functions in modulating specific types of cell death, dependent on autophagy and ROS-mediated signaling. Although autophagic pathways have been widely explored as a strategy for overcoming mycobacterial escape from phagosomal maturation, excessive activation of autophagy, and the resulting cell death (caused by a robust increase in ROS generation), did not apparently directly impact host defenses in Mtb-*Δeis*-infected cells.

Autophagy is a well-organized homeostatic cellular process responsible for the removal of damaged organelles and the elimination of intracellular pathogens [5]. Induction of autophagy is critical to the eradication of Mtb from murine and human macrophages [7], [8]. Recent reports have emphasized the role of autophagy in host defense against human tuberculosis caused by Mtb [36]. Prolonged or excessive autophagy can result in non-apoptotic type II programmed cell death [10]. We recently reported that mycobacterial BCG cell wall induced autophagic cell death in radiosensitized cancer cells [34]. Indeed, it is known that several cytokines, including TNF-α, can activate autophagy pathways [5]. Thus, because they have been shown to be potent inducers of cytokine production [37], it is possible that mycobacterial proteins and/or other cell components increase the activation of autophagy by inducing the production of TNF-α. In our recent study, we found that mycobacterial LpqH can trigger the activation of autophagy [38]. Additionally, various mycobacterial components, including ESAT6 [39], PE_PGRS 33 [40], and nuoG [41], have been reported in modulating host cell death, i.e., apoptosis or necrosis. Moreover, a recent report showed that Mtb mutation of *nuoG* or *KatG* leads to ROS accumulation in phagosomes, with subsequent induction of host cell apoptosis [42]. However, the genetic basis of mycobacterial induction of autophagy-dependent cell death in normal macrophages has not been characterized. Macrophages that died after Mtb-*Δeis* infection displayed morphological features of autophagic (type 2) cell death, characterized by the accumulation of autophagic vacuoles (autophagosomes) in the cytoplasm [43]. Massive autophagic vacuolization may be the consequence of a failed attempt by Mtb to adapt to its cellular environment, which ultimately results in cell death [43].

Macrophages infected with Mtb-*Δeis* at a relatively low MOI (5–10) displayed higher rates of CICD and autophagy activation than did cells infected with wild-type or complemented strains of Mtb H37Rv. These data partly correlate with the previous finding that infection with Mtb H37Rv at the same MOI slightly increased macrophage cytotoxicity over control levels [44]. It has also been shown that attenuated strains of mycobacteria at an MOI≤10 trigger TNF-α-induced apoptosis, which is associated with innate host defenses against intracellular mycobacteria [45]. Macrophages infected with Mtb-*Δeis* showed a modest, but significant, increase in the rate of apoptosis, as assessed by the TUNEL assay. Additionally, we observed no prominent sign of necrosis [46], such as intracellular vesicular swelling, rupture of plasma membranes, or dilation of cytoplasmic organelles, in macrophages infected with Mtb-*Δeis*. Thus, our data show that Mtb Eis is involved in the control of a novel type of cell death, characterized by massive autophagic vacuolization. This type of cell death was modulated by inhibitors of autophagy: 3-MA (see Fig. 4).

After showing that Eis is involved in autophagy-dependent cell death, we considered the possibility that Eis may affect the intracellular survival of bacteria. We previously reported that an *eis* deletion mutant of Mtb had no growth defect in human monocytic U937 cells or in mice [16]. The current study confirms our previous findings [16] that an *eis* deletion mutant of H37Rv multiplies at a rate similar to WT or complemented strains in the lungs and spleen of infected mice (see Fig. 6D). In contrast, a recent study reported that deletion of *eis* reduced the growth of the clinical Mtb strain TB294 in Mono Mac 6 cells [18]. This clinical strain was found to express 20-fold higher levels of Eis than H37Rv [18]. This discrepancy may be the result of strain-specific differences in the production of Eis and/or the use of different host cells [18]. Recent findings showed that an *eis* promoter mutation that increases Eis expression conferred resistance to kanamycin in clinical Mtb strains, by increasing its acetylation and inactivation [17]. We thus suggest that overproduction of Eis may enable some clinical Mtb strains to modulate autophagy and cell death, especially those with *eis* promoter mutations. It will be interesting to determine whether clinical strains overproducing Eis exhibit altered intracellular growth and disease outcomes through subversion of autophagy, cell death, and host defense.

Activation of an exacerbated inflammatory response during Mtb-*Δeis* infection may explain the lack of effect on bacterial elimination despite the induction of autophagy. Indeed, our previous [16] and current studies showed that inflammatory responses are profoundly up-regulated in Mtb-*Δeis*-infected monocytes/macrophages. Here, proinflammatory cytokine production was negatively regulated by autophagy activation in Mtb-*Δeis*-infected macrophages (Fig. S2). Despite the potential contribution of autophagy to this inflammatory balance, increased ROS and subsequent organelle damage by Mtb-*Δeis* infection may trigger an amplifying positive feedback loop and in so doing induce massive autophagy and cell death. If production of pro-inflammatory cytokines and chemokines during mycobacterial infection is excessive or inappropriate, it may hinder protective immunity and exacerbate the pathology [47].

Our data show that significant up-regulation of ROS production (for which NOX and mitochondria are largely responsible) is required for Mtb-*Δeis* to increase macrophage inflammatory and autophagic responses, which are normally controlled by Eis. These findings partially agree with our previous studies showing that NOX2-dependent ROS generation played a key role in TLR2-dependent inflammatory signaling and cathelicidin production in macrophages [28]. Selective autophagic degradation of catalase and subsequent ROS accumulation cause lipid membrane damage and autophagic cell death, indicating the complex nature of the relationship between ROS and non-apoptotic programmed cell death [48]. Additionally, overproduction of ROS contributed to CICD in macrophages treated with lipopolysaccharide and the pan-caspase inhibitor, Z-VAD [48]. At the molecular level, “Toll/IL-1 receptor (TIR) domain-containing adaptor-inducing IFN-β” (TRIF) and “receptor-interacting protein 1” (RIP-1) operate upstream of ROS production and are involved in inducing autophagy and CICD [49]. In starvation-induced autophagy, ROS serve as signaling molecules that induce autophagy and regulate cysteine protease HsAtg4 [50]. Moreover, previous studies showed that activation of TLRs or Fcγ receptors induced autophagy through NADPH oxidase-derived ROS [30].

Regarding the signaling pathways linking ROS and cell death, our data provide evidence for the involvement of JNK signaling in macrophages infected with Mtb-*Δeis*. Recent studies have shown that JNK pathways contribute to the induction of non-canonical autophagy by activating Atg7 [51]. Additionally, other studies showed that increased oxidative stress results in the induction of endoplasmic reticulum stress, which, in turn, can lead to autophagy and cell death through activation of a JNK/p38 signaling pathway [52]. Moreover, JNK signaling has been shown to play an important role in autophagic cell death [53]. We found that the activation of JNK was required for Mtb-*Δeis*-induced ROS generation and cell death. Thus, it appears that oxidative stress and JNK/SAPK constitute a positive feedback loop that contributes to the induction of cell death with autophagy by Mtb-*Δeis*.

Given the specific pathologic events that occur in Mtb-*Δeis*-infected macrophages, several mechanisms could explain the observed excessive autophagy and resulting cell death. First, excessive ROS generation (for which NOX and mitochondria are primarily responsible), may contribute to increased activation of autophagy. Data generated using ROS inhibitors and NOX2-deficient mice show that excessive ROS generation is responsible for the induction of autophagy and inflammation by Mtb-*Δeis*. The marked induction of autophagy by Mtb-*Δeis* may be attributed to the expected need for increased protein/organelle turnover in injured cells undergoing oxidative stress, such as those with damaged mitochondria [54]. Second, our findings suggest that Eis regulates a key player in host innate immunity through its *N*-acetyltransferase domain. This idea is supported by the observations that Mtb-*Δeis*-mediated ROS generation and inflammatory cytokine production were inhibited by pretreatment with Eis and realized in an *N*-acetyltransferase domain-dependent manner (see Fig. 7). Finally, the phenotype of Mtb-*Δeis*-infected macrophages may depend on their activation state. When macrophages were primed using interferon-γ and lipopolysaccharide prior to exposure to Mtb-*Δeis*, they showed a significant decrease in overall cell death, but a concurrent increase in the rate of apoptosis (data not shown). These data indicate that the activation of macrophages may alter their mechanism of cell death during subsequent Mtb-*Δeis* infection. Thus, excessive activation of autophagy appears to play an important role in cell death, although cell death with autophagy does not affect the ability of host cells to efficiently eliminate invading bacteria.

Our data provide evidence that Eis plays an essential role in regulating both the early generation of ROS and inflammatory responses in macrophages. These activities are dependent on the acetyltransferase moiety of Eis. Previously, we reported that Eis is a member of the GCN5-related family of *N*-acetyltransferases, as determined through bioinformatics analyses [16]. Members of this family of proteins are involved in a variety of activities, ranging from transcriptional activation to antibiotic resistance [55]. The well-characterized effector YopJ from *Yersinia* spp. acetylated critical serine and threonine residues in the activation loop of MAPKK6, thereby blocking its phosphorylation [56]. This resulted in the inhibition of MAPK and nuclear factor-κB signaling and, thus, the innate immune responses to *Yersinia* infection [56], [57]. The current data suggest that the mycobacterial effector Eis regulates eukaryotic cell function through the direct modification of target proteins, effected by its *N*-acetyltransferase domain.

Together, our results provide novel insights into the roles of mycobacterial Eis in controlling and suppressing host inflammatory responses and cell survival/death, which it achieves by modulating ROS-dependent JNK activation. Excessive activation of autophagy was shown to cause cell death, as well as inefficient bacterial clearance, in macrophages infected with Mtb-*Δeis*. Eis itself regulated oxidative stress and inflammation through its acetyltransferase domain. Our present characterization of the mycobacterial effector Eis as a modulator of autophagy and cell death presents a previously unknown paradigm for understanding host-pathogen interactions in mycobacterial infection.

## Materials and Methods

### Bacterial Strains and Recombinant Eis Protein

Mtb-WT, Mtb-*Δeis*, and Mtb-*c-eis* strains were generated and used in these experiments. The *eis* gene was disrupted in H37Rv by means of a two-step gene replacement strategy using a pMJ10 allelic exchange vector (*ts ori*M; *sac*B counterselection; Kan^R^, Gent^R^) as described previously [58]. A vector was constructed that contained the *eis* gene disrupted by a hygromycin cassette (*eis*:*hyg*/pMJ10). This vector (5 µg) was introduced into electrocompetent Mtb H37Rv cells. Transformants were first selected by growth on 7H10-OADC-Tween 80 plates containing hygromycin B (50 µg/mL) at 37°C for 3–4 weeks. Individual antibiotic-resistant colonies were selected and subcultured onto fresh plates. Several clones were then picked and grown in 50 mL of 7H9-ADC broth containing hygromycin B at 37°C for 48 h. Cells from the broth cultures were then diluted in 7H9-ADC broth and plated on 2% sucrose-7H10-OADC-hyg and incubated at 39°C for 3–4 weeks. The double-resistant (suc^R^/hyg^R^) clones were selected and shown, by Southern blotting, to be *Δeis* mutants (data not shown). The H37Rv*Δeis* mutant was complemented using an integration vector (pMV306) containing a single copy of the *eis* gene (mycobacterial integration vector; integrates into the *att*B site; Kan^R^) [59]. To obtain purified Eis protein, N-terminally His-tagged Eis was induced, harvested and purified from, *E. coli* expression strain BL-21 DE-3 pLysS, as described by Samuel *et al*. [16] following standard protocols recommended by Novagen. Mtb strains were grown as described previously [13]. The bacterial cultures were divided into 1-mL aliquots in cryovials and stored at −70°C prior to use. Representative vials were thawed, and viable CFUs were counted on Middlebrook 7H10 agar. Single-cell suspensions of mycobacteria were prepared as described previously [60].

### Ethics Statement

All animal procedures were approved by the Institutional Animal Care and Use Committees of Yonsei University Health System and Chungnam National University. All animal experiments were performed in accordance with Korean Food and Drug Administration (KFDA) guidelines.

### Mice and Cells

For *in vivo* experiments, pathogen-free female C57BL/6 mice, aged 5–6 weeks, purchased from Japan SLC Inc. (Shijuoka, Japan) were maintained under barrier conditions in a BL-3 biohazard animal room at Yonsei University Medical Research Center. Animals were fed a sterile commercial mouse diet and water *ad libitum*. NOX2 (C57BL/6 background) mice were kindly provided by Y. S. Bae (Iwha University, Seoul). Mice used as a source of cells for *in vitro* experiments were housed in specific pathogen-free conditions. Those used in individual experiments were age- and sex-matched mice (and 5–8 weeks of age). BMDMs were isolated and then differentiated by growth for 5–7 days in medium containing M-CSF (25 µg/mL; R&D), as described previously [13]. RAW 264.7 cells were maintained in Dulbecco's modified Eagle's medium (DMEM) containing 10% fetal bovine serum, as described previously [13]. Human THP-1 (ATCC TIB-202) monocytic cells were grown in RPMI 1640/GlutaMAX, supplemented with 10% FBS [8]. Cells were treated with 20 nM PMA (Sigma-Aldrich, St. Louis, MO) for 24 h to induce their differentiation into macrophage-like cells and then washed three times with PBS.

### Mtb Infection and Bacterial Counts *In Vitro* and *In Vivo*

*In vitro* macrophage infection was performed as described previously [13]. Briefly, cells were infected with mycobacteria at different MOIs and incubated for 4 h at 37°C in a 5% CO_2_ atmosphere. After allowing time for phagocytosis, cells were washed four times with fresh PBS to remove extracellular bacteria and then incubated with complete DMEM without antibiotics. As controls, cultures of uninfected macrophages (UI) were maintained under the same conditions. The infection rates for the three strains were approximately 35–45% when BMDMs were infected at an MOI of 5. Rates of infection were increased in infected macrophages when the MOI was increased. There was no significant difference in infection rates between the three strains.

To test the capacities of the Mtb-WT, Mtb-*Δeis*, and Mtb-*c-eis* strains to survive intracellularly, BMDMs were infected with each strain at MOIs of 1 and 5. Then, 4 h later, cells were washed with PBS three times, and the majority of extracellular bacteria (>99%) were removed, as determined through staining with auramine-rhodamine (Merck, Darmstadt, Germany). After washing, the cells were incubated in fresh medium for a further 3 days. They were then lysed in autoclaved distilled water to allow intracellular bacteria to be collected [8]. The lysates were then re-suspended and sonicated for 5 min in a preheated 37°C water bath sonicator (Elma, Singen, Germany). Aliquots of the resulting sonicates were serially diluted in 7H9 broth, plated separately on 7H10 agar plates, and incubated at 37°C in 5% CO_2_ for 12 d. Colony counting was then performed in triplicate.

Mice were challenged by aerosol exposure with Mtb-WT, Mtb-*Δeis*, or Mtb-*c-eis* using an inhalation device (Glas-Col, Terre Haute, IN, USA) calibrated to deliver approximately 50 bacteria into the lungs. Five mice per group were sacrificed at 4 weeks post-challenge, and bacteria in lung and spleen homogenates were counted. Numbers of viable bacteria in lung/spleen were determined by plating serial dilutions of whole organ homogenates on Middlebrook 7H11 agar (Difco, Detroit, MI, USA). Colonies were counted after 3–4 weeks of incubation at 37°C.

### Reagents, DNA, Abs, and Transfection

DPI (a NOX inhibitor), NAC (an antioxidant), catalase, and z-VAD-fmk (a pan-caspase inhibitor) were purchased from Calbiochem (San Diego, CA, USA). 3-MA, tiron (a commercial deflocculant), and DAPI were purchased from Sigma. DMSO (Sigma) was added to cultures at a concentration of 0.1% (v/v) as a solvent control (SC). The plasmid that encoded EGFP-LC3 [20] was a gift from Tamotsu Yoshimori (Osaka University, Japan). pCMV-Eis-WT and pCMV-Eis-*Δ*AT constructs were created by subcloning the whole *eis* gene (WT), or an acetyltransferase domain-deletion mutant (lacking the sequence encoding residues 61–137 of the 402-amino-acid Eis protein) from pET21a. This was achieved by cutting at the *Bam*HI and *Sac*I restriction sites and then ligating the resulting inserts into the pCMV-Tag1 mammalian expression vector (Stratagene Co., USA).

Anti-LC3 antibodies (Abs) used for Western blotting and immunofluorescence analysis were purchased from Novus Biologicals and MBL International (Woburn, MA, USA), respectively. Anti-rabbit IgG-Alexa488 and IgG-TRITC, and anti-mouse IgG-Cy2, were purchased from Jackson Immunoresearch (West Grove, PA, USA). siRNAs specific for *mBeclin 1* (sc-29798), *mAtg5* (sc-41446), and *mJNK1* (sc-29381), each a pool of five target-specific 19–25 nt siRNAs, were purchased from Santa Cruz Biotechnology (Santa Cruz, CA, USA). Cells were transfected with plasmids and/or siRNAs using Lipofectamine 2000 (Invitrogen, Carlsbad, CA, USA) according to the manufacturer's protocol.

### Measurement of ROS Production

Intracellular ROS levels were measured by DCFH-DA and DHE assays as described previously [61]. Briefly, BMDMs were differentiated in culture dishes and infected with bacterial strains (MOI = 10) for 30 min. Cells were then incubated with either DCFH-DA (5 µM) or DHE (10 µM; Molecular Probes) for 30 min at 37°C in 5% CO_2_ and then washed with Krebs-Hepes buffer (for DHE staining) or HBSS (for DCFH-DA staining). Total intracellular levels of ROS were determined by FACS analyses of the oxidative conversion of cell-permeable DCFH-DA (Molecular Probes) to fluorescent DHE (Molecular Probes), using the FACSCanto II system (Becton Dickinson, San Jose, CA, USA).

A mitochondrion-specific hydroethidine-derivative fluorescent dye (MitoSOX; M36008; Calbiochem) was used to determine relative mitochondrial O_2_^−^ levels in BMDMs. Cells were incubated for 30 min in PBS containing 5 µM MitoSOX. They were then washed twice and analyzed using the FACSCanto II system. All FACS data were collected using 50,000 to 100,000 cells and analyzed using FlowJo software (Tree Star, Ashland, OR, USA).

### Cell Viability and Apoptosis Assays

Cell viability was assessed by PI staining and then examined by fluorescence microscopy or flow cytometric analysis. Trypan blue-stained cells were counted using a ViCell counter (Beckman Coulter, Fullerton, CA, USA). Apoptosis was examined by TdT-mediated dUTP Nick-End Labeling (TUNEL; Promega), according to the manufacturer's instructions. Labeled cells were examined under a laser-scanning confocal microscope (model LSM 510; Zeiss). Each condition was assayed in triplicate, and at least 200 cells per well were counted. To analyze *in vivo* cell death, single-cell suspensions were prepared in RPMI 1640 medium by passing cell populations through a nylon mesh with 50 µm pores and were subjected to further analysis.

### Western Blotting, RT-PCR and ELISA

Treated BMDMs were processed for analysis by sandwich ELISA, Western blotting, and RT-PCR as described previously [2]. For Western blot analysis, primary Abs were diluted 1∶1000. Membranes were developed using a chemiluminescent reagent (ECL; Pharmacia-Amersham, Freiburg, Germany) and subsequently exposed to film (Pharmacia-Amersham). Supernatant TNF-α and IL-6 levels were measured by sandwich ELISA using Duoset Ab pairs (Pharmingen, San Diego, CA, USA) [2].

To provide RNA for RT-PCR analysis, paraffin-embedded tissue sections were first deparaffinized in octane [62]. After vigorous vortexing, 150 µL of methanol were added. Samples were vortexed again and the tissue was pelleted by centrifugation (10,000×*g*, 2 min). Supernatants were removed, and the remaining tissue was vacuum-dried for 20 min. Next, pellets were resuspended in digestion buffer (20 mM Tris-HCl, pH 7.6, 0.5% N-laurylsarcosine, 1 M guanidine thiocyanate, 25 mM 2-mercaptoethanol) containing proteinase K (5 mg/mL; Sigma). After overnight digestion at 55°C, RNA was extracted using TRIzol (Invitrogen) according to the manufacturer's instructions. For quantitative RT-PCR analysis was performed by using SYBR Green (Molecular Probes) PCR core reagents (Applied Biosystems), and transcript levels were quantified by using an ABI 7900 Sequence Detection System (Applied Biosystems). The mean value of triplicate reactions was normalized against the mean value of β-actin. Primers were used at 400 nM.

### Autophagy Analysis

Autophagosome formation was measured by LC3 punctate staining, as described previously [8]. To quantitate autophagy, we used fluorescence microscopy to count the percentages of GFP-LC3-positive autophagic vacuoles in transfected cells or the numbers of endogenous LC3 punctate dots in primary cells. Each condition was assayed in triplicate, and at least 200 cells per well were counted. LC3 conjugation was evaluated by Western blot analysis using an antibody raised to LC3-I/II.

### Transmission Electron Microscopy

Infected and stimulated RAW 264.7 macrophages were washed with PBS and then fixed with 3% formaldehyde, 2% glutaraldehyde in 0.1 M sodium cacodylate buffer (pH 7.4) for 1 h. They were then post-fixed in 1% osmium tetroxide, 0.5% potassium ferricyanide in cacodylate buffer for 1 h; embedded in straight resin; and cured at 80°C for 24 h. Ultrathin sections (70–80 nm), cut using an ultramicrotome (RMC MT6000-XL), were stained with uranyl acetate and lead citrate and examined using a Tecnai G2 Spirit Twin transmission electron microscope (FEI Company, USA) and a JEM ARM 1300S High Voltage electron microscope (JEOL, Japan).

### Immunofluorescence

Immunofluorescence analysis was performed as described previously [8]. Briefly, cells were fixed with 4% paraformaldehyde in PBS at 4°C for 10 min and permeabilized with 0.01% Triton X-100 in PBS for 10 min. Cultures were then stained for 2 h at room temperature with primary antibodies, including rabbit anti-mouse LC3 (1∶400; MBL International). After washing, to remove excess primary antibody, cultures were then incubated for 1 h at room temperature with an anti-rabbit IgG-Alexa488 secondary antibody (Jackson Immunoresearch). Nuclei were stained by incubation with DAPI for 5 min. Slides were examined using a laser-scanning confocal microscope (model LSM 510; Zeiss).

### Statistical Analyses

Data obtained from independent experiments (presented as mean±SD) were analyzed by the paired Student's *t*-test with Bonferroni correction or analysis of variance (for multiple comparisons). A *p* value<0.05 was deemed to indicate statistical significance.

### Accession Numbers

The GenBank accession number for the *eis* gene is AF144099.

## Supporting Information

Figure S1 Autophagic vesicles are increased in macrophages infected with Mtb-*Δeis*, but not in cells infected with Mtb-WT or Mtb-*c-eis*. (A and B) Formation of GFP-LC3 vacuoles (dots) was determined in RAW 264.7 cells transfected with GFP-LC3 cDNA. Transfected cells were infected with Mtb-WT, Mtb-*Δeis*, or Mtb-*c-eis* (MOI = 10) for 24 h (A) or Mtb-*Δeis* (MOI = 10) for 24 h in the presence or absence of 3-MA (B). *Top*, representative immunofluorescence images; *bottom*, percentage of GFP-LC3 cells with punctae. (C) Co-localization of autophagosomes (endogenous LC3, red) and lysosomes (lamp-1, green) was increased in Mtb-*Δeis*-infected BMDMs. Data are representative of three separate experiments. Scale bars: 10 µm. (D) BMDMs were infected with Mtb-*Δeis* (MOI = 10) for 24 h in the presence or absence of 3-MA (10 mM) or Baf-A1 (100 nM). Quantitation of the percentages of cells with LC3 punctae. Each condition was assayed in triplicate, and at least 250 cells per well were counted. ***p<0.001, vs. Mtb-WT-infected condition (A); SC (B and D). UI, uninfected; SC, solvent control (0.1% distilled water (B), 0.1% DMSO (D)).
(0.66 MB TIF)

Figure S2 Activation of autophagy negatively impacts the secretion of proinflammatory cytokines by Mtb-*Δeis*-infected macrophages. RAW 264.7 cells transfected with siRNAs specific for *Beclin-1* (siBec-1) or *Atg5* (siAtg5) were infected with Mtb-WT, Mtb-*Δeis*, or Mtb-*c-eis* (MOI = 10) for 24 h. Supernatants were assessed by ELISA for levels of TNF-α and IL-6. Data are presented as the mean±SD of five experiments. ***p<0.001, vs. Mtb-WT-infected condition. UI, uninfected.
(0.12 MB TIF)

Figure S3 Reactive nitrogen species are not involved in the elevation of ROS generation in Mtb-*Δeis*-infected macrophages. BMDMs were infected with Mtb-*Δeis* (MOI = 10) in the presence or absence of L-NAME (0.1, 1, 5 mM) or L-NMMA (0.1, 1, 5 mM). Cells were stained with DHE (for superoxide) or DCFH-DA (for H_2_O_2_) and subjected to flow cytometry analysis. Data represent densitometric analyses (mean±SD) of three separate experiments. UI, uninfected; SC, solvent control.
(0.07 MB TIF)

Figure S4 Intracellular ROS and NOX2 are required for autophagy and proinflammatory responses in Mtb-*Δeis*-infected macrophages. (A) RAW 264.7 cells transfected with GFP-LC3 cDNA were infected with Mtb-*Δeis* (MOI = 10) in the presence or absence of DPI (10 µM), NAC (20 mM), catalase (Cat, 1 mU/mL), or tiron (5 mM). Formation of GFP-LC3 vacuoles (dots) was determined in transfected cells, and at least 250 cells per well were counted. *Left:* representative immunofluorescence images; *right:* percentage of LC3-punctated cells. (B) BMDMs from WT and NOX2-KO mice were infected with Mtb-WT, Mtb-*Δeis*, or Mtb-*c*-*eis* (MOI = 10). After 30 min, ROS production (DHE staining) was determined by flow cytometry (*left*). Quantitative analysis of ROS generation in WT- and NOX2-deficient BMDMs (*right*). Data represent the mean±SD of three independent experiments. (C) BMDMs from WT and NOX2 KO mice were treated with rapamycin (Rapa; 20 µg/mL) or staurosporine (STS; 500 nM), or nutrient-starved (Starv; maintained in HBSS) for 8 h. Numbers of LC3-punctated cells (counted manually) are shown. Data are presented as the mean±SD of at least three separate experiments, each performed in triplicate. (D) BMDMs from WT and NOX2 KO mice were infected with Mtb-WT, Mtb-*Δeis*, or Mtb-*c*-*eis* for 6 h and then subjected to RT-PCR analysis. A gel representative of three independent replicates is shown. *** *p*<0.001, vs. SC (A); WT mice (B). UI, uninfected; SC, solvent control (0.1% DMSO).
(0.38 MB TIF)

Figure S5 Enhanced cell death in Mtb-*Δeis*-infected macrophages is regulated by autophagic pathways. (A) BMDMs were infected with Mtb-WT, Mtb-*Δeis*, or Mtb-*c*-*eis* at the indicated MOIs for 4 h, washed to remove unbound mycobacteria, and then incubated in complete DMEM at 37°C in 5% CO_2_ for the indicated periods of time. Cells were stained with PI and then examined by fluorescence microscopy. (B) Cell death was determined in RAW 264.7 cells transfected with specific siRNA for *beclin-1*, *atg5,* or non-specific scrambled siRNA (siNS) before infection with Mtb-WT, Mtb-*Δeis*, or Mtb-*c*-*eis*, as described in the Materials and Methods. After 36 h, cells were stained with PI and examined by fluorescence microscopy, as described in the Materials and Methods. Transfection efficiency was assessed by RT-PCR (*inset*). (C) Experimental conditions were identical to those outlined in panel A. Cell viability was assessed by trypan blue staining. Data are presented as the mean±SD of three separate experiments, each performed in duplicate. *p<0.05, ***p<0.001, vs. Mtb-WT-infected condition (A).
(0.16 MB TIF)
