# Supplementary figures and images for: Mycobacterium tuberculosis Eis Regulates Autophagy, Inflammation, and Cell Death through Redox-dependent Signaling
Source: PLoS Pathog. 2010 Dec 16;6(12):e1001230. doi: 10.1371/journal.ppat.1001230 (PMC3002989; doi:10.1371/journal.ppat.1001230)

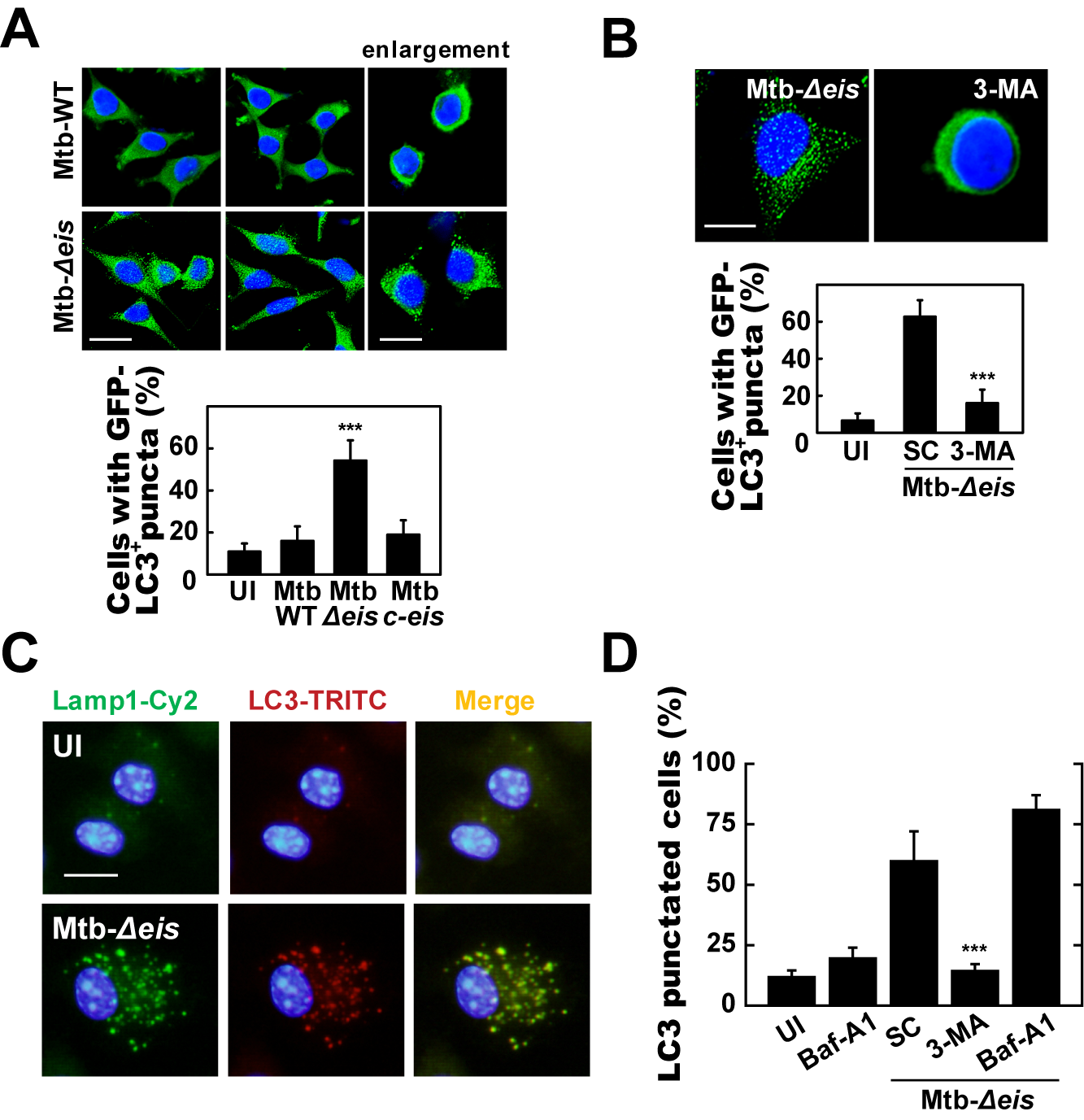

Supplement: Figure S1 — Autophagic vesicles are increased in macrophages infected with Mtb-Δeis, but not in cells infected with Mtb-WT or Mtb-c-eis. (A and B) Formation of GFP-LC3 vacuoles (dots) was determined in RAW 264.7 cells transfected with GFP-LC3 cDNA. Transfected cells were infected with Mtb-WT, Mtb-Δeis, or Mtb-c-eis (MOI = 10) for 24 h (A) or Mtb-Δeis (MOI = 10) for 24 h in the presence or absence of 3-MA (B). Top, representative immunofluorescence images; bottom, percentage of GFP-LC3 cells with punctae. (C) Co-localization of autophagosomes (endogenous LC3, red) and lysosomes (lamp-1, green) was increased in Mtb-Δeis-infected BMDMs. Data are representative of three separate experiments. Scale bars: 10 µm. (D) BMDMs were infected with Mtb-Δeis (MOI = 10) for 24 h in the presence or absence of 3-MA (10 mM) or Baf-A1 (100 nM). Quantitation of the percentages of cells with LC3 punctae. Each condition was assayed in triplicate, and at least 250 cells per well were counted. ***p<0.001, vs. Mtb-WT-infected condition (A); SC (B and D). UI, uninfected; SC, solvent control (0.1% distilled water (B), 0.1% DMSO (D)). (0.66 MB TIF) [file ppat.1001230.s001.tif]

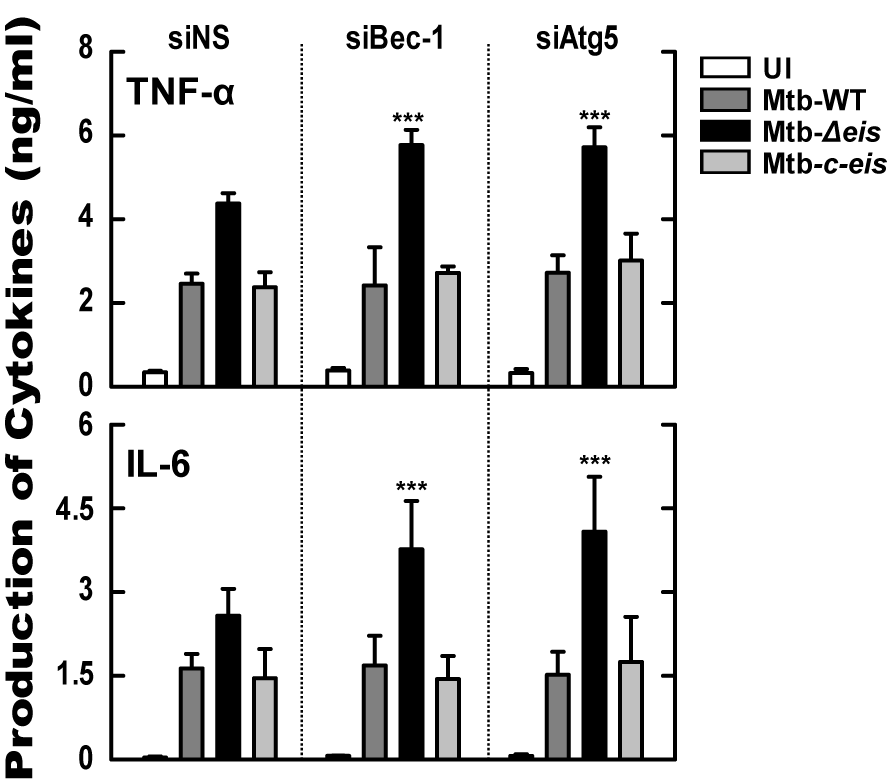

Supplement: Figure S2 — Activation of autophagy negatively impacts the secretion of proinflammatory cytokines by Mtb-Δeis-infected macrophages. RAW 264.7 cells transfected with siRNAs specific for Beclin-1 (siBec-1) or Atg5 (siAtg5) were infected with Mtb-WT, Mtb-Δeis, or Mtb-c-eis (MOI = 10) for 24 h. Supernatants were assessed by ELISA for levels of TNF-α and IL-6. Data are presented as the mean±SD of five experiments. ***p<0.001, vs. Mtb-WT-infected condition. UI, uninfected. (0.12 MB TIF) [file ppat.1001230.s002.tif]

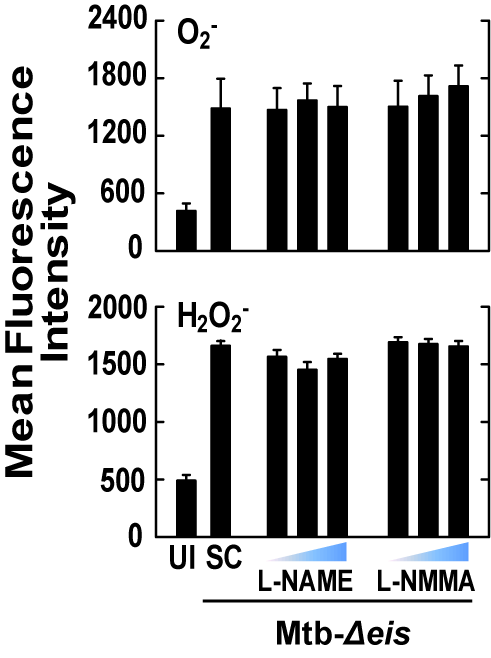

Supplement: Figure S3 — Reactive nitrogen species are not involved in the elevation of ROS generation in Mtb-Δeis-infected macrophages. BMDMs were infected with Mtb-Δeis (MOI = 10) in the presence or absence of L-NAME (0.1, 1, 5 mM) or L-NMMA (0.1, 1, 5 mM). Cells were stained with DHE (for superoxide) or DCFH-DA (for H2O2) and subjected to flow cytometry analysis. Data represent densitometric analyses (mean±SD) of three separate experiments. UI, uninfected; SC, solvent control. (0.07 MB TIF) [file ppat.1001230.s003.tif]

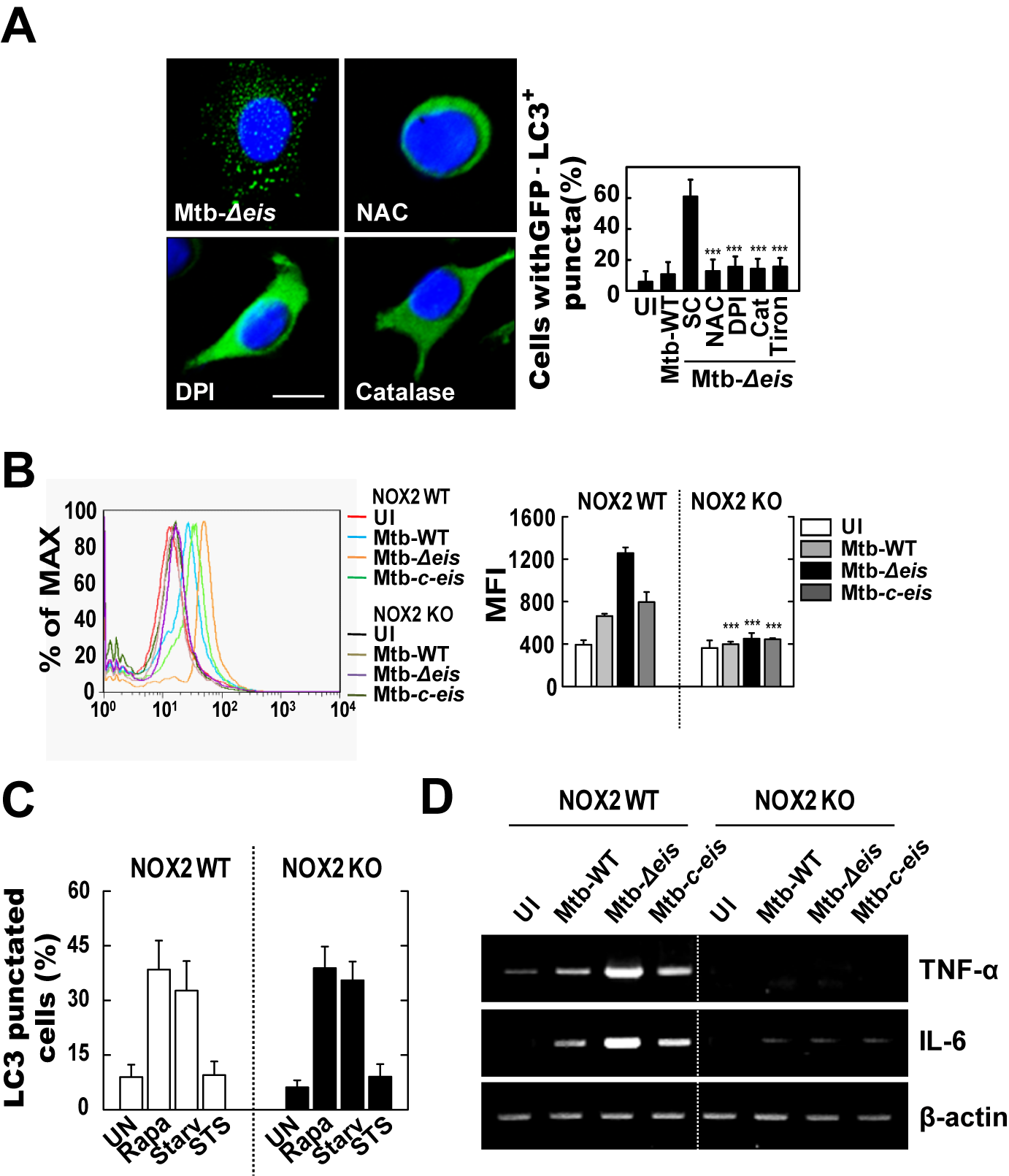

Supplement: Figure S4 — Intracellular ROS and NOX2 are required for autophagy and proinflammatory responses in Mtb-Δeis-infected macrophages. (A) RAW 264.7 cells transfected with GFP-LC3 cDNA were infected with Mtb-Δeis (MOI = 10) in the presence or absence of DPI (10 µM), NAC (20 mM), catalase (Cat, 1 mU/mL), or tiron (5 mM). Formation of GFP-LC3 vacuoles (dots) was determined in transfected cells, and at least 250 cells per well were counted. Left: representative immunofluorescence images; right: percentage of LC3-punctated cells. (B) BMDMs from WT and NOX2-KO mice were infected with Mtb-WT, Mtb-Δeis, or Mtb-c-eis (MOI = 10). After 30 min, ROS production (DHE staining) was determined by flow cytometry (left). Quantitative analysis of ROS generation in WT- and NOX2-deficient BMDMs (right). Data represent the mean±SD of three independent experiments. (C) BMDMs from WT and NOX2 KO mice were treated with rapamycin (Rapa; 20 µg/mL) or staurosporine (STS; 500 nM), or nutrient-starved (Starv; maintained in HBSS) for 8 h. Numbers of LC3-punctated cells (counted manually) are shown. Data are presented as the mean±SD of at least three separate experiments, each performed in triplicate. (D) BMDMs from WT and NOX2 KO mice were infected with Mtb-WT, Mtb-Δeis, or Mtb-c-eis for 6 h and then subjected to RT-PCR analysis. A gel representative of three independent replicates is shown. *** p<0.001, vs. SC (A); WT mice (B). UI, uninfected; SC, solvent control (0.1% DMSO). (0.38 MB TIF) [file ppat.1001230.s004.tif]

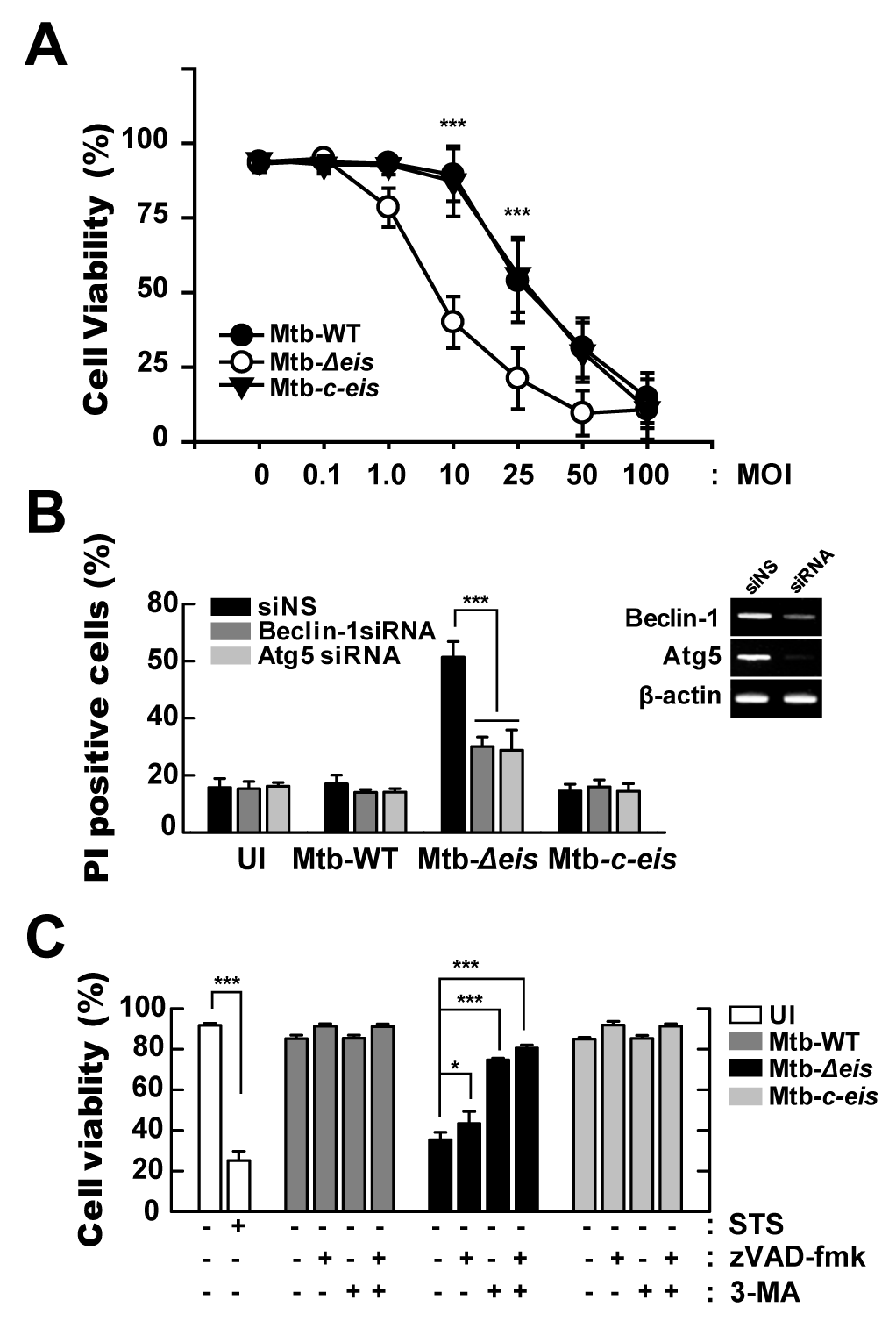

Supplement: Figure S5 — Enhanced cell death in Mtb-Δeis-infected macrophages is regulated by autophagic pathways. (A) BMDMs were infected with Mtb-WT, Mtb-Δeis, or Mtb-c-eis at the indicated MOIs for 4 h, washed to remove unbound mycobacteria, and then incubated in complete DMEM at 37°C in 5% CO2 for the indicated periods of time. Cells were stained with PI and then examined by fluorescence microscopy. (B) Cell death was determined in RAW 264.7 cells transfected with specific siRNA for beclin-1, atg5, or non-specific scrambled siRNA (siNS) before infection with Mtb-WT, Mtb-Δeis, or Mtb-c-eis, as described in the Materials and Methods. After 36 h, cells were stained with PI and examined by fluorescence microscopy, as described in the Materials and Methods. Transfection efficiency was assessed by RT-PCR (inset). (C) Experimental conditions were identical to those outlined in panel A. Cell viability was assessed by trypan blue staining. Data are presented as the mean±SD of three separate experiments, each performed in duplicate. *p<0.05, ***p<0.001, vs. Mtb-WT-infected condition (A). (0.16 MB TIF) [file ppat.1001230.s005.tif]
